# Supplementary figures and images for: Microbiota composition effect on immunotherapy outcomes in colorectal cancer patients: A systematic review
Source: PLoS One. 2024 Jul 24;19(7):e0307639. doi: 10.1371/journal.pone.0307639 (PMC11268651; doi:10.1371/journal.pone.0307639)

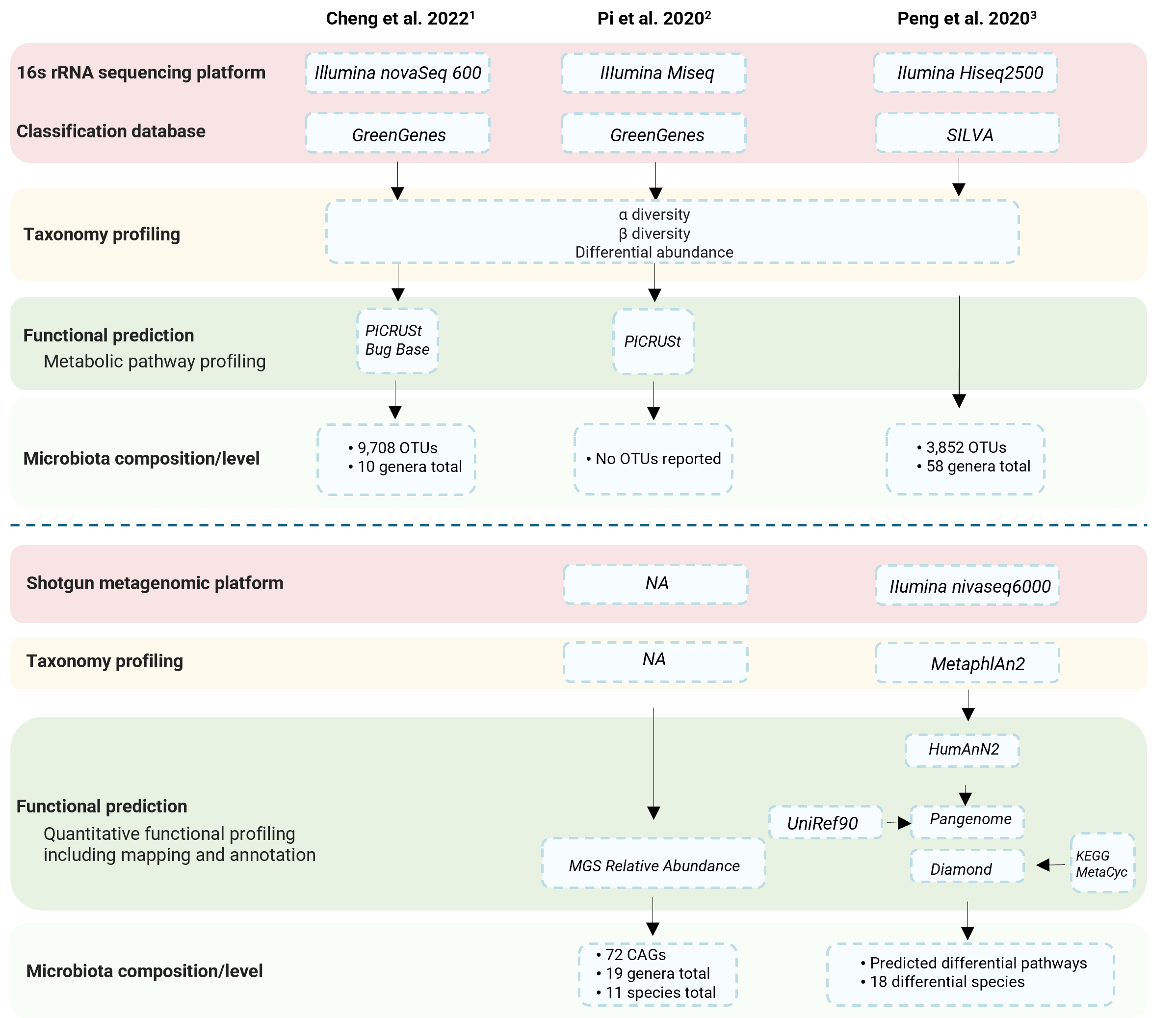

Supplement: S1 Fig — (TIF) [file pone.0307639.s001.tif]
