# Supplementary material for: Microbiota composition effect on immunotherapy outcomes in colorectal cancer patients: A systematic review
Source: PLoS One. 2024 Jul 24;19(7):e0307639. doi: 10.1371/journal.pone.0307639 (PMC11268651; doi:10.1371/journal.pone.0307639)
Supplement: S1 Table — (PDF) [file pone.0307639.s002.pdf]

Table S1. Updated search for “The role of microbiota in immunotherapy outcomes in colorectal cancer patients”

**Source and search date**	**Search string**	**Results and notes**
**PubMed**<br><br>(NLM)<br><br>**Coverage:**<br><br>from database inception to<br><br>To<br><br>2023-07-18	(("Digestive System Diseases"[Mesh] OR "Digestive System Disease*"[Title/Abstract] OR "Digestive System Disorder*"[Title/Abstract] OR "Digestive System Neoplasms"[Mesh] OR "Digestive System Neoplasm*"[Title/Abstract] OR "Cancer of Digestive System*"[Title/Abstract] OR "Cancer of the Digestive System*"[Title/Abstract] OR "Digestive System Cancer*"[Title/Abstract] OR "Gastrointestinal Diseases"[Mesh] OR "Gastrointestinal Disease*"[Title/Abstract] OR "Gastrointestinal Disorder*"[Title/Abstract] OR "Functional Gastrointestinal Disorder*"[Title/Abstract] OR "Gastrointestinal Neoplasms"[Mesh] OR "Gastrointestinal Neoplasm*"[Title/Abstract] OR "Cancer of Gastrointestinal Tract*"[Title/Abstract] OR "Gastrointestinal Tract Cancer*"[Title/Abstract] OR "Cancer of the Gastrointestinal Tract"[Title/Abstract] OR "Gastrointestinal Cancer*"[Title/Abstract] OR "Intestinal Diseases"[Mesh] OR "Intestinal Disease*"[Title/Abstract] OR "Intestinal Neoplasms"[Mesh] OR "Intestinal Neoplasm*"[Title/Abstract] OR "Intestines Neoplasm*"[Title/Abstract] OR "Intestine Neoplasm*"[Title/Abstract] OR "Intestine cancer*"[Title/Abstract] OR "Cancer of Intestine*"[Title/Abstract] OR "Intestines Cancer*"[Title/Abstract] OR "Intestine Cancer*"[Title/Abstract] OR "Cancer of the Intestine*"[Title/Abstract] OR "Intestinal Cancer*"[Title/Abstract] OR "Colorectal Neoplasms"[Mesh] OR "Colorectal Neoplasm*"[Title/Abstract] OR "Colorectal Tumor*"[Title/Abstract] OR "Colorectal Cancer*"[Title/Abstract] OR "Colorectal Carcinoma*"[Title/Abstract] OR "Colonic Diseases"[Mesh] OR "Colonic Disease*"[Title/Abstract] OR "Colonic Neoplasms"[Mesh] OR "Colonic Neoplasm*"[Title/Abstract] OR "Colon Neoplasm*"[Title/Abstract] OR "Cancer of Colon"[Title/Abstract] OR "Cancer of the Colon"[Title/Abstract] OR "Colon Cancer*"[Title/Abstract] OR "Colonic Cancer*"[Title/Abstract] OR "Colon Adenocarcinoma*"[Title/Abstract] OR "Colitis-Associated Neoplasms"[Mesh] OR "Colitis Associated Neoplasm*"[Title/Abstract] OR "Colitis-Associated Neoplasm*"[Title/Abstract] OR "Colitis-Associated Colorectal Cancer*"[Title/Abstract] OR "Colitis Associated Colorectal Cancer*"[Title/Abstract] OR "Colitis-Associated Cancer*"[Title/Abstract] OR "Colitis Associated Cancer*"[Title/Abstract] OR "Colitis-Associated Colon Cancer*"[Title/Abstract] OR "Colitis Associated Colon Cancer*"[Title/Abstract] OR "Sigmoid Neoplasms"[Mesh] OR "Sigmoid Neoplasm*"[Title/Abstract] OR	**Results:**<br><br>**2067**<br><br>**Notes:**<br><br>All keywords are searched in the fields: “title” and “abstract” and in MeSH when available.<br><br>The search is limited to English studies only.

	"Sigmoid Colon Neoplasm*"[Title/Abstract] OR "Sigmoid Cancer*"[Title/Abstract] OR "Sigmoidal Cancer*"[Title/Abstract] OR "Sigmoid Colon Cancer*"[Title/Abstract] OR "Cancer of Sigmoid"[Title/Abstract] OR "Cancer of the Sigmoid"[Title/Abstract] OR "Intestinal Polyps"[Mesh] OR "Intestinal Polyps*"[Title/Abstract] OR "Colonic Polyps"[Mesh] OR "Colonic Polyp*"[Title/Abstract]) AND ("Gastrointestinal Microbiome*"[MeSH] OR "Gastrointestinal Microbiome*"[Title/Abstract] OR "Gut Microbiome*"[Title/Abstract] OR "Gut Microflora"[Title/Abstract] OR "Gut Microbiota*"[Title/Abstract] OR "Gastrointestinal Flora"[Title/Abstract] OR "Gut Flora"[Title/Abstract] OR "Gastrointestinal Microbiota*"[Title/Abstract] OR "Gastrointestinal Microbial Communit*"[Title/Abstract] OR "Gastrointestinal Microflora"[Title/Abstract] OR "Gastric Microbiome*"[Title/Abstract] OR "Intestinal Microbiome*"[Title/Abstract] OR "Intestinal Microbiota*"[Title/Abstract] OR "Intestinal Microflora"[Title/Abstract] OR "Intestinal Flora"[Title/Abstract] OR "Enteric Bacteria*"[Title/Abstract] OR "Dysbiosis"[Mesh] OR "Dysbios*"[Title/Abstract] OR "Dys-symbios*"[Title/Abstract] OR "Dysbacterios*"[Title/Abstract] OR "Disbacterios*"[Title/Abstract] OR "Sequence Analysis, DNA"[Mesh] OR "Sequence Analyses, DNA"[Title/Abstract] OR "DNA Sequence Analys*"[Title/Abstract] OR "DNA Sequence Determination*"[Title/Abstract] OR "DNA Sequencing"[Title/Abstract] OR "DNA Barcoding, Taxonomic"[Mesh] OR "Taxonomic DNA Barcoding*"[Title/Abstract] OR "Phylogenetic DNA Barcoding*"[Title/Abstract] OR "Taxonomic DNA Barcode*"[Title/Abstract] OR "Phylogenetic DNA Barcode*"[Title/Abstract] OR "Whole Genome Sequencing"[Mesh] OR "Whole Genome Sequencing*"[Title/Abstract] OR "Complete Genome Sequencing*"[Title/Abstract] OR "Whole Exome Sequencing"[Mesh] OR "Whole Exome Sequencing*"[Title/Abstract] OR "Complete Exome Sequencing*"[Title/Abstract] OR "Whole Transcriptome Sequencing*"[Title/Abstract] OR "Complete Transcriptome Sequencing*"[Title/Abstract] OR "High-Throughput Nucleotide Sequencing"[Mesh] OR "High Throughput Nucleotide Sequencing"[Title/Abstract] OR "Next-Generation Sequencing"[Title/Abstract] OR "Next Generation Sequencing"[Title/Abstract] OR "Illumina Sequencing"[Title/Abstract] OR "Ion Torrent Sequencing"[Title/Abstract] OR "Ion Proton Sequencing"[Title/Abstract] OR "Deep Sequencing"[Title/Abstract] OR "High-Throughput RNA Sequencing"[Title/Abstract] OR "High Throughput RNA Sequencing"[Title/Abstract] OR "Massively-Parallel Sequencing"[Title/Abstract] OR "Massively Parallel Sequencing"[Title/Abstract] OR "Pyrosequencing"[Title/Abstract] OR "High-Throughput Sequencing"[Title/Abstract] OR "High Throughput Sequencing"[Title/Abstract] OR "High-Throughput DNA Sequencing"[Title/Abstract] OR "High Throughput DNA Sequencing"[Title/Abstract])) AND ("Immunotherapy"[Mesh] OR	

	"Immunotherp*"[Title/Abstract] OR "Biological Therapy"[Mesh] OR "Biological Therap*"[Title/Abstract] OR "Biologic Therap*"[Title/Abstract] OR "Biotherap*"[Title/Abstract] OR "Immune Checkpoint Inhibitors"[Mesh] OR "Immune Checkpoint Inhibitor"[Title/Abstract] OR "Immune Checkpoint Blocker*"[Title/Abstract] OR "Immune Checkpoint Blockade"[Title/Abstract] OR "Immune Checkpoint Inhibition"[Title/Abstract] OR "PD-L1 Inhibitor*"[Title/Abstract] OR "PD L1 Inhibitor*"[Title/Abstract] OR "Programmed Death-Ligand 1 Inhibitor*"[Title/Abstract] OR "Programmed Death Ligand 1 Inhibitor*"[Title/Abstract] OR "PD-1-PD-L1 Blockade"[Title/Abstract] OR "PD 1 PD L1 Blockade"[Title/Abstract] OR "CTLA-4 Inhibitor*"[Title/Abstract] OR "CTLA 4 Inhibitor*"[Title/Abstract] OR "Cytotoxic T-Lymphocyte-Associated Protein 4 Inhibitor*"[Title/Abstract] OR "Cytotoxic T Lymphocyte Associated Protein 4 Inhibitor*"[Title/Abstract] OR "PD-1 Inhibitor*"[Title/Abstract] OR "PD 1 Inhibitor*"[Title/Abstract] OR "Programmed Cell Death Protein 1 Inhibitor*"[Title/Abstract] OR "Pembrolizumab"[Title/Abstract] OR "Keytruda"[Title/Abstract] OR "Nivolumab"[Mesh] OR "Nivolumab"[Title/Abstract] OR "Opdivo"[Title/Abstract] OR "Ipilimumab"[Mesh] OR "Ipilimumab"[Title/Abstract] OR "Yervoy"[Title/Abstract] OR "Immunomodulation"[Mesh] OR "Immunomodulation*"[Title/Abstract] OR "Immunomodulatory Therap*"[Title/Abstract] OR "Immunotherapy, Adoptive"[Mesh:NoExp] OR "Adoptive Transfer"[Mesh:NoExp])	
**Scopus**<br>(Elsevier)<br>**Coverage:**<br>from database inception<br>To<br>2023-07-18	TITLE-ABS-KEY("Digestive System Disease*" OR "Digestive System Disorder*" OR "Digestive System Neoplasm*" OR "Cancer of Digestive System*" OR "Cancer of the Digestive System*" OR "Digestive System Cancer*" OR "Gastrointestinal Disease*" OR "Gastrointestinal Disorder*" OR "Functional Gastrointestinal Disorder*" OR "Gastrointestinal Neoplasm*" OR "Cancer of Gastrointestinal Tract*" OR "Gastrointestinal Tract Cancer*" OR "Cancer of the Gastrointestinal Tract" OR "Gastrointestinal Cancer*" OR "Intestinal Disease*" OR "Intestinal Neoplasm*" OR "Intestines Neoplasm*" OR "Intestine Neoplasm*" OR "Intestine cancer*" OR "Cancer of Intestine*" OR "Intestines Cancer*" OR "Intestine Cancer*" OR "Cancer of the Intestine*" OR "Intestinal Cancer*" OR "Colorectal Neoplasm*" OR "Colorectal Tumor*" OR "Colorectal Cancer*" OR "Colorectal Carcinoma*" OR "Colonic Disease*" OR "Colonic Neoplasm*" OR "Colon Neoplasm*" OR "Cancer of Colon" OR "Cancer of the Colon" OR "Colon Cancer*" OR "Colonic Cancer*" OR "Colon Adenocarcinoma*" OR "Colitis Associated Neoplasm*" OR "Colitis-Associated Neoplasm*" OR "Colitis-Associated Colorectal Cancer*" OR "Colitis Associated Colorectal Cancer*" OR "Colitis-Associated Cancer*" OR "Colitis Associated Cancer*" OR "Colitis-Associated Colon Cancer*" OR "Colitis Associated Colon Cancer*" OR "Sigmoid Neoplasm*" OR "Sigmoid Colon Neoplasm*" OR	**Results:**<br>**1,341**<br>**Notes:**<br>All keywords are searched in the fields: “title”, “abstract” and “keywords” (here marked with “TITLE-ABS-KEY “).<br>No Subject headings or thesaurus

	"Sigmoid Cancer*" OR "Sigmoidal Cancer*" OR "Sigmoid Colon Cancer*" OR "Cancer of Sigmoid" OR "Cancer of the Sigmoid" OR "Intestinal Polyps*" OR "Colonic Polyp*") AND TITLE-ABS-KEY("Gastrointestinal Microbiome*" OR "Gut Microbiome*" OR "Gut Microflora" OR "Gut Microbiota*" OR "Gastrointestinal Flora" OR "Gut Flora" OR "Gastrointestinal Microbiota*" OR "Gastrointestinal Microbial Communit*" OR "Gastrointestinal Microflora" OR "Gastric Microbiome*" OR "Intestinal Microbiome*" OR "Intestinal Microbiota*" OR "Intestinal Microflora" OR "Intestinal Flora" OR "Enteric Bacteria*" OR "Dysbios*" OR "Dys-symbios*" OR "Dysbacterios*" OR "Disbacterios*" OR "Sequence Analyses, DNA" OR "DNA Sequence Analys*" OR "DNA Sequence Determination*" OR "DNA Sequencing" OR "Taxonomic DNA Barcoding*" OR "Phylogenetic DNA Barcoding*" OR "Taxonomic DNA Barcode*" OR "Phylogenetic DNA Barcode*" OR "Whole Genome Sequencing*" OR "Complete Genome Sequencing*" OR "Whole Exome Sequencing*" OR "Complete Exome Sequencing*" OR "Whole Transcriptome Sequencing*" OR "Complete Transcriptome Sequencing*" OR "High Throughput Nucleotide Sequencing" OR "Next-Generation Sequencing" OR "Next Generation Sequencing" OR "Illumina Sequencing" OR "Ion Torrent Sequencing" OR "Ion Proton Sequencing" OR "Deep Sequencing" OR "High-Throughput RNA Sequencing" OR "High Throughput RNA Sequencing" OR "Massively-Parallel Sequencing" OR "Massively Parallel Sequencing" OR "Pyrosequencing" OR "High-Throughput Sequencing" OR "High Throughput Sequencing" OR "High-Throughput DNA Sequencing" OR "High Throughput DNA Sequencing") AND TITLE-ABS-KEY("Immunotherp*" OR "Biological Therap*" OR "Biologic Therap*" OR "Biotherap*" OR "Immune Checkpoint Inhibitor" OR "Immune Checkpoint Blocker*" OR "Immune Checkpoint Blockade" OR "Immune Checkpoint Inhibition" OR "PD-L1 Inhibitor*" OR "PD L1 Inhibitor*" OR "Programmed Death-Ligand 1 Inhibitor*" OR "Programmed Death Ligand 1 Inhibitor*" OR "PD-1-PD-L1 Blockade" OR "PD 1 PD L1 Blockade" OR "CTLA-4 Inhibitor*" OR "CTLA 4 Inhibitor*" OR "Cytotoxic T-Lymphocyte-Associated Protein 4 Inhibitor*" OR "Cytotoxic T Lymphocyte Associated Protein 4 Inhibitor*" OR "PD-1 Inhibitor*" OR "PD 1 Inhibitor*" OR "Programmed Cell Death Protein 1 Inhibitor*" OR "Pembrolizumab" OR "Keytruda" OR "Nivolumab" OR "Opdivo" OR "Ipilimumab" OR "Yervoy" OR "Immunomodulation*" OR "Immunomodulatory Therap*")	available in Scopus.<br><br>The search is limited to English studies only
**Embase**<br><br>(Elsevier, all sources included)<br><br>All sources	'digestive system disease*':ab,ti OR 'digestive system disorder*':ab,ti OR 'digestive system neoplasm*':ab,ti OR 'cancer of digestive system*':ab,ti OR 'cancer of the digestive system*':ab,ti OR 'digestive system cancer*':ab,ti OR 'gastrointestinal disease*':ab,ti OR 'gastrointestinal disorder*':ab,ti OR 'functional gastrointestinal disorder*':ab,ti OR 'gastrointestinal neoplasm*':ab,ti OR 'cancer of gastrointestinal tract*':ab,ti OR 'gastrointestinal tract cancer*':ab,ti OR 'cancer of the gastrointestinal tract':ab,ti OR 'gastrointestinal cancer*':ab,ti OR 'intestinal disease*':ab,ti OR 'intestinal neoplasm*':ab,ti OR 'intestines neoplasm*':ab,ti OR	**Results:**<br><br>**3,278**<br><br>**Notes:**<br><br>All keywords are searched in

**Coverage:** from database inception<br><br>To<br><br>2023-07-18	'intestine neoplasm*':ab,ti OR 'cancer of intestine*':ab,ti OR 'intestines cancer*':ab,ti OR 'intestine cancer*':ab,ti OR 'cancer of the intestine*':ab,ti OR 'intestinal cancer*':ab,ti OR 'colorectal neoplasm*':ab,ti OR 'colorectal tumor*':ab,ti OR 'colorectal cancer*':ab,ti OR 'colorectal carcinoma*':ab,ti OR 'colonic disease*':ab,ti OR 'colonic neoplasm*':ab,ti OR 'colon neoplasm*':ab,ti OR 'cancer of colon':ab,ti OR 'cancer of the colon':ab,ti OR 'colon cancer*':ab,ti OR 'colonic cancer*':ab,ti OR 'colon adenocarcinoma*':ab,ti OR 'colitis associated neoplasm*':ab,ti OR 'colitis-associated neoplasm*':ab,ti OR 'colitis-associated colorectal cancer*':ab,ti OR 'colitis associated colorectal cancer*':ab,ti OR 'colitis-associated cancer*':ab,ti OR 'colitis associated cancer*':ab,ti OR 'colitis-associated colon cancer*':ab,ti OR 'colitis associated colon cancer*':ab,ti OR 'sigmoid neoplasm*':ab,ti OR 'sigmoid colon neoplasm*':ab,ti OR 'sigmoid cancer*':ab,ti OR 'sigmoidal cancer*':ab,ti OR 'sigmoid colon cancer*':ab,ti OR 'cancer of sigmoid':ab,ti OR 'cancer of the sigmoid':ab,ti OR 'intestinal polyps*':ab,ti OR 'colonic polyp*':ab,ti<br><br>OR<br><br>'gastrointestinal disease'/exp OR 'intestine tumor'/exp OR 'gastrointestinal tumor'/de OR 'intestine cancer'/exp OR 'gastrointestinal cancer'/de OR 'digestive system carcinoma'/de OR 'digestive system cancer'/de OR 'digestive system tumor'/de OR 'colorectal tumor'/exp OR 'colon disease'/de OR 'large intestine disease'/exp OR 'colitis-associated cancer'/exp OR 'large intestine tumor'/exp OR 'intestine polyp'/exp<br><br>AND<br><br>'gastrointestinal microbiome*':ab,ti OR 'gut microbiome*':ab,ti OR 'gut microflora':ab,ti OR 'gut microbiota*':ab,ti OR 'gastrointestinal flora':ab,ti OR 'gut flora':ab,ti OR 'gastrointestinal microbiota*':ab,ti OR 'gastrointestinal microbial communit*':ab,ti OR 'gastrointestinal microflora':ab,ti OR 'gastric microbiome*':ab,ti OR 'intestinal microbiome*':ab,ti OR 'intestinal microbiota*':ab,ti OR 'intestinal microflora':ab,ti OR 'intestinal flora':ab,ti OR 'enteric bacteria*':ab,ti OR 'dysbios*':ab,ti OR 'dys-symbios*':ab,ti OR 'dysbacterios*':ab,ti OR 'disbacterios*':ab,ti OR 'sequence analyses, dna':ab,ti OR 'dna sequence analys*':ab,ti OR 'dna sequence determination*':ab,ti OR 'dna sequencing':ab,ti OR 'taxonomic dna barcoding*':ab,ti OR 'phylogenetic dna barcoding*':ab,ti OR 'taxonomic dna barcode*':ab,ti OR 'phylogenetic dna barcode*':ab,ti OR 'whole genome sequencing*':ab,ti OR 'complete genome sequencing*':ab,ti OR 'whole exome sequencing*':ab,ti OR 'complete exome sequencing*':ab,ti OR 'whole transcriptome sequencing*':ab,ti OR 'complete transcriptome sequencing*':ab,ti OR 'high throughput nucleotide sequencing':ab,ti OR 'next-generation sequencing':ab,ti OR 'next generation sequencing':ab,ti OR 'illumina sequencing':ab,ti OR 'ion torrent sequencing':ab,ti OR 'ion proton sequencing':ab,ti OR 'deep sequencing':ab,ti OR 'high-throughput rna sequencing':ab,ti OR 'high throughput rna sequencing':ab,ti OR 'massively-	the fields: "title" and "abstract" and "keywords" (here marked with "TI" and "AB") and in the Embase Emtree when available (here marked with "mj" or "exp").<br><br>The search is limited to English studies only.

	parallel sequencing':ab,ti OR 'massively parallel sequencing':ab,ti OR 'pyrosequencing':ab,ti OR 'high-throughput sequencing':ab,ti OR 'high throughput sequencing':ab,ti OR 'high-throughput dna sequencing':ab,ti OR 'high throughput dna sequencing':ab,ti<br><br>OR<br><br>'intestine flora'/exp OR 'dysbiosis'/exp OR 'dna sequencing'/exp OR 'dna barcoding'/exp OR 'whole genome sequencing'/exp OR 'whole exome sequencing'/exp OR 'high throughput sequencing'/exp<br><br>AND<br><br>'immunotherp*':ab,ti OR 'biological therap*':ab,ti OR 'biologic therap*':ab,ti OR 'biotherap*':ab,ti OR 'immune checkpoint inhibitor':ab,ti OR 'immune checkpoint blocker*':ab,ti OR 'immune checkpoint blockade':ab,ti OR 'immune checkpoint inhibition':ab,ti OR 'pd-l1 inhibitor*':ab,ti OR 'pd l1 inhibitor*':ab,ti OR 'programmed death-ligand 1 inhibitor*':ab,ti OR 'programmed death ligand 1 inhibitor*':ab,ti OR 'pd-1-pd-l1 blockade':ab,ti OR 'pd 1 pd l1 blockade':ab,ti OR 'ctla-4 inhibitor*':ab,ti OR 'ctla 4 inhibitor*':ab,ti OR 'cytotoxic t-lymphocyte-associated protein 4 inhibitor*':ab,ti OR 'cytotoxic t lymphocyte associated protein 4 inhibitor*':ab,ti OR 'pd-1 inhibitor*':ab,ti OR 'pd 1 inhibitor*':ab,ti OR 'programmed cell death protein 1 inhibitor*':ab,ti OR 'pembrolizumab':ab,ti OR 'keytruda':ab,ti OR 'nivolumab':ab,ti OR 'opdivo':ab,ti OR 'ipilimumab':ab,ti OR 'yervoy':ab,ti OR 'immunomodulation*':ab,ti OR 'immunomodulatory therap*':ab,ti<br><br>OR<br><br>'immunotherapy'/de OR 'active immunotherapy'/exp OR 'antibody therapy'/exp OR 'cancer immunotherapy'/exp OR 'biological therapy'/de OR 'immune checkpoint inhibitor'/exp OR 'nivolumab'/exp OR 'ipilimumab'/exp OR 'immunomodulation'/exp	
**Web of Science- Core Collection**<br><br>(Clarivate)<br><br>**Coverage:** from database inception to<br><br>To<br><br>2023-07-18	"Digestive System Disease*" OR "Digestive System Disorder*" OR "Digestive System Neoplasm*" OR "Cancer of Digestive System*" OR "Cancer of the Digestive System*" OR "Digestive System Cancer*" OR "Gastrointestinal Disease*" OR "Gastrointestinal Disorder*" OR "Functional Gastrointestinal Disorder*" OR "Gastrointestinal Neoplasm*" OR "Cancer of Gastrointestinal Tract*" OR "Gastrointestinal Tract Cancer*" OR "Cancer of the Gastrointestinal Tract" OR "Gastrointestinal Cancer*" OR "Intestinal Disease*" OR "Intestinal Neoplasm*" OR "Intestines Neoplasm*" OR "Intestine Neoplasm*" OR "Intestine cancer*" OR "Cancer of Intestine*" OR "Intestines Cancer*" OR "Intestine Cancer*" OR "Cancer of the Intestine*" OR "Intestinal Cancer*" OR "Colorectal Neoplasm*" OR "Colorectal Tumor*" OR "Colorectal Cancer*" OR "Colorectal Carcinoma*" OR "Colonic Disease*" OR	**Results:**<br><br>**270**<br><br>**Notes:**<br><br>All keywords are searched in the fields: “title”, “abstract” and “keywords” (here marked

	"Colonic Neoplasm*" OR "Colon Neoplasm*" OR "Cancer of Colon" OR "Cancer of the Colon" OR "Colon Cancer*" OR "Colonic Cancer*" OR "Colon Adenocarcinoma*" OR "Colitis Associated Neoplasm*" OR "Colitis-Associated Neoplasm*" OR "Colitis-Associated Colorectal Cancer*" OR "Colitis Associated Colorectal Cancer*" OR "Colitis-Associated Cancer*" OR "Colitis Associated Cancer*" OR "Colitis-Associated Colon Cancer*" OR "Colitis Associated Colon Cancer*" OR "Sigmoid Neoplasm*" OR "Sigmoid Colon Neoplasm*" OR "Sigmoid Cancer*" OR "Sigmoidal Cancer*" OR "Sigmoid Colon Cancer*" OR "Cancer of Sigmoid" OR "Cancer of the Sigmoid" OR "Intestinal Polyps*" OR "Colonic Polyp*" (Topic) and "Gastrointestinal Microbiome*" OR "Gut Microbiome*" OR "Gut Microflora" OR "Gut Microbiota*" OR "Gastrointestinal Flora" OR "Gut Flora" OR "Gastrointestinal Microbiota*" OR "Gastrointestinal Microbial Communit*" OR "Gastrointestinal Microflora" OR "Gastric Microbiome*" OR "Intestinal Microbiome*" OR "Intestinal Microbiota*" OR "Intestinal Microflora" OR "Intestinal Flora" OR "Enteric Bacteria*" OR "Dysbios*" OR "Dys-symbios*" OR "Dysbacterios*" OR "Disbacterios*" OR "Sequence Analyses, DNA" OR "DNA Sequence Analys*" OR "DNA Sequence Determination*" OR "DNA Sequencing" OR "Taxonomic DNA Barcoding*" OR "Phylogenetic DNA Barcoding*" OR "Taxonomic DNA Barcode*" OR "Phylogenetic DNA Barcode*" OR "Whole Genome Sequencing*" OR "Complete Genome Sequencing*" OR "Whole Exome Sequencing*" OR "Complete Exome Sequencing*" OR "Whole Transcriptome Sequencing*" OR "Complete Transcriptome Sequencing*" OR "High Throughput Nucleotide Sequencing" OR "Next-Generation Sequencing" OR "Next Generation Sequencing" OR "Illumina Sequencing" OR "Ion Torrent Sequencing" OR "Ion Proton Sequencing" OR "Deep Sequencing" OR "High-Throughput RNA Sequencing" OR "High Throughput RNA Sequencing" OR "Massively-Parallel Sequencing" OR "Massively Parallel Sequencing" OR "Pyrosequencing" OR "High-Throughput Sequencing" OR "High Throughput Sequencing" OR "High-Throughput DNA Sequencing" OR "High Throughput DNA Sequencing" (Topic) and "Immunotherp*" OR "Biological Therap*" OR "Biologic Therap*" OR "Biotherap*" OR "Immune Checkpoint Inhibitor" OR "Immune Checkpoint Blocker*" OR "Immune Checkpoint Blockade" OR "Immune Checkpoint Inhibition" OR "PD-L1 Inhibitor*" OR "PD L1 Inhibitor*" OR "Programmed Death-Ligand 1 Inhibitor*" OR "Programmed Death Ligand 1 Inhibitor*" OR "PD-1-PD-L1 Blockade" OR "PD 1 PD L1 Blockade" OR "CTLA-4 Inhibitor*" OR "CTLA 4 Inhibitor*" OR "Cytotoxic T-Lymphocyte-Associated Protein 4 Inhibitor*" OR "Cytotoxic T Lymphocyte Associated Protein 4 Inhibitor*" OR "PD-1 Inhibitor*" OR "PD 1 Inhibitor*" OR "Programmed Cell Death Protein 1 Inhibitor*" OR "Pembrolizumab" OR "Keytruda" OR "Nivolumab" OR "optico" OR "Ipilimumab" OR "yerkoy" OR "Immunomodulation*" OR "Immunomodulatory Therap*" (Topic) and English (Languages)	with “TOPIC “).<br><br>No Subject headings or thesaurus available in Web of Science.<br><br>The search is limited to English studies only

**Cochrane Library**<br>(Cochrane coloboration)<br>**Coverage:** from database inception to<br>To:<br>2023-07-18	("Digestive System Disease*" OR "Digestive System Disorder*" OR "Digestive System Neoplasm*" OR "Cancer of Digestive System*" OR "Cancer of the Digestive System*" OR "Digestive System Cancer*" OR "Gastrointestinal Disease*" OR "Gastrointestinal Disorder*" OR "Functional Gastrointestinal Disorder*" OR "Gastrointestinal Neoplasm*" OR "Cancer of Gastrointestinal Tract*" OR "Gastrointestinal Tract Cancer*" OR "Cancer of the Gastrointestinal Tract" OR "Gastrointestinal Cancer*" OR "Intestinal Disease*" OR "Intestinal Neoplasm*" OR "Intestines Neoplasm*" OR "Intestine Neoplasm*" OR "Intestine cancer*" OR "Cancer of Intestine*" OR "Intestines Cancer*" OR "Intestine Cancer*" OR "Cancer of the Intestine*" OR "Intestinal Cancer*" OR "Colorectal Neoplasm*" OR "Colorectal Tumor*" OR "Colorectal Cancer*" OR "Colorectal Carcinoma*" OR "Colonic Disease*" OR "Colonic Neoplasm*" OR "Colon Neoplasm*" OR "Cancer of Colon" OR "Cancer of the Colon" OR "Colon Cancer*" OR "Colonic Cancer*" OR "Colon Adenocarcinoma*" OR "Colitis Associated Neoplasm*" OR "Colitis-Associated Neoplasm*" OR "Colitis-Associated Colorectal Cancer*" OR "Colitis Associated Colorectal Cancer*" OR "Colitis-Associated Cancer*" OR "Colitis Associated Cancer*" OR "Colitis-Associated Colon Cancer*" OR "Colitis Associated Colon Cancer*" OR "Sigmoid Neoplasm*" OR "Sigmoid Colon Neoplasm*" OR "Sigmoid Cancer*" OR "Sigmoidal Cancer*" OR "Sigmoid Colon Cancer*" OR "Cancer of Sigmoid" OR "Cancer of the Sigmoid" OR "Intestinal Polyps*" OR "Colonic Polyp*"):ti,ab,kw (Word variations have been searched)<br>OR<br>MeSH descriptor: [Colorectal Neoplasms] explode all trees<br>OR MeSH descriptor: [Intestinal Neoplasms] explode all trees<br>OR MeSH descriptor: [Gastrointestinal Neoplasms] this term only<br>OR MeSH descriptor: [Digestive System Neoplasms] this term only<br>OR MeSH descriptor: [Digestive System Diseases] this term only<br>OR MeSH descriptor: [Gastrointestinal Diseases] this term only<br>OR MeSH descriptor: [Intestinal Diseases] explode all trees<br>OR MeSH descriptor: [Colonic Diseases] explode all trees<br>OR MeSH descriptor: [Rectal Diseases] explode all trees<br>OR MeSH descriptor: [Colitis-Associated Neoplasms] explode all trees<br>OR MeSH descriptor: [Intestinal Polyps] explode all trees	**Results:**<br>**62**<br>**Notes:**<br>All keywords are searched in the fields: “title” and “abstract” and in MeSH (when available)<br>The Cochrane Library doesn’t have language filter.

	AND<br><br>("Gastrointestinal Microbiome*" OR "Gut Microbiome*" OR "Gut Microflora" OR "Gut Microbiota*" OR "Gastrointestinal Flora" OR "Gut Flora" OR "Gastrointestinal Microbiota*" OR "Gastrointestinal Microbial Communit*" OR "Gastrointestinal Microflora" OR "Gastric Microbiome*" OR "Intestinal Microbiome*" OR "Intestinal Microbiota*" OR "Intestinal Microflora" OR "Intestinal Flora" OR "Enteric Bacteria*" OR "Dysbios*" OR "Dys-symbios*" OR "Dysbacterios*" OR "Disbacterios*" OR "Sequence Analyses, DNA" OR "DNA Sequence Analys*" OR "DNA Sequence Determination*" OR "DNA Sequencing" OR "Taxonomic DNA Barcoding*" OR "Phylogenetic DNA Barcoding*" OR "Taxonomic DNA Barcode*" OR "Phylogenetic DNA Barcode*" OR "Whole Genome Sequencing*" OR "Complete Genome Sequencing*" OR "Whole Exome Sequencing*" OR "Complete Exome Sequencing*" OR "Whole Transcriptome Sequencing*" OR "Complete Transcriptome Sequencing*" OR "High Throughput Nucleotide Sequencing" OR "Next-Generation Sequencing" OR "Next Generation Sequencing" OR "Illumina Sequencing" OR "Ion Torrent Sequencing" OR "Ion Proton Sequencing" OR "Deep Sequencing" OR "High-Throughput RNA Sequencing" OR "High Throughput RNA Sequencing" OR "Massively-Parallel Sequencing" OR "Massively Parallel Sequencing" OR "Pyrosequencing" OR "High-Throughput Sequencing" OR "High Throughput Sequencing" OR "High-Throughput DNA Sequencing" OR "High Throughput DNA Sequencing"):ti,ab,kw (Word variations have been searched)<br><br>OR<br><br>MeSH descriptor: [Gastrointestinal Microbiome] explode all trees<br><br>OR MeSH descriptor: [Dysbiosis] this term only<br><br>OR MeSH descriptor: [Sequence Analysis, DNA] explode all trees<br><br>OR MeSH descriptor: [DNA Barcoding, Taxonomic] explode all trees<br><br>OR MeSH descriptor: [Whole Genome Sequencing] explode all trees<br><br>OR MeSH descriptor: [Whole Exome Sequencing] explode all trees<br><br>OR MeSH descriptor: [Sequence Analysis] explode all trees<br><br>OR MeSH descriptor: [High-Throughput Nucleotide Sequencing] explode all trees<br><br>AND	

	("Immunotherp*" OR "Biological Therap*" OR "Biologic Therap*" OR "Biotherap*" OR "Immune Checkpoint Inhibitor" OR "Immune Checkpoint Blocker*" OR "Immune Checkpoint Blockade" OR "Immune Checkpoint Inhibition" OR "PD-L1 Inhibitor*" OR "PD L1 Inhibitor*" OR "Programmed Death-Ligand 1 Inhibitor*" OR "Programmed Death Ligand 1 Inhibitor*" OR "PD-1-PD-L1 Blockade" OR "PD 1 PD L1 Blockade" OR "CTLA-4 Inhibitor*" OR "CTLA 4 Inhibitor*" OR "Cytotoxic T-Lymphocyte-Associated Protein 4 Inhibitor*" OR "Cytotoxic T Lymphocyte Associated Protein 4 Inhibitor*" OR "PD-1 Inhibitor*" OR "PD 1 Inhibitor*" OR "Programmed Cell Death Protein 1 Inhibitor*" OR "Pembrolizumab" OR "Keytruda" OR "Nivolumab" OR "Opdivo" OR "Ipilimumab" OR "Yervoy" OR "Immunomodulation*" OR "Immunomodulatory Therap*"):ti,ab,kw (Word variations have been searched)<br><br>OR<br><br>MeSH descriptor: [Immunotherapy] explode all trees<br>OR MeSH descriptor: [Biological Therapy] this term only<br>OR MeSH descriptor: [Immune Checkpoint Inhibitors] explode all trees<br>OR MeSH descriptor: [Antibodies, Monoclonal, Humanized] explode all trees	
**Records identified in the search**		**7,018**
**Unique records identified after de-duplication in Covidence**		**5,132**
