# Supplementary material for: Microbiota composition effect on immunotherapy outcomes in colorectal cancer patients: A systematic review
Source: PLoS One. 2024 Jul 24;19(7):e0307639. doi: 10.1371/journal.pone.0307639 (PMC11268651; doi:10.1371/journal.pone.0307639)
Supplement: S3 Table — (PDF) [file pone.0307639.s004.pdf]

Table S3. Overview of bioinformatics databases utilized for taxonomic identification in the reviewed studies.

**Comparison Parameters**	**SILVA database**(1–3)	**GreenGenes database**(4–8)	**MetaPhlAn2 database**(9–11)
**Database Size**	9 million reference sequences (Over 8 million small sub-units and over 1 million large sub-units)	202,421 reference sequences	over 17,000 reference sequences (>_1 million clade-specific marker genes from)
**Reference Genome Sequences**	Curated from the ARB-SILVA database, which includes sequences from the EMBL database	A curated set of 16S rRNA sequences, mainly NCBI data	Most likely curated set sequences from Public databases
**Taxonomic Coverage**	Archaea, bacteria, and eukaryotes	Archaea and bacteria	Archaea, bacteria, eukaryotes and viruses
**Level of Taxonomic Resolution**	Species-level	Species-level	Strain level
**Update Frequency**	Approximately twice a year	Infrequently updated, the latest major update was in May 2013	Frequently updated, metaphlAnV4.0.6 (2022/2023-latest).
**Precision and Accuracy**	High accuracy: it employs SINA to maintain high alignment accuracy.	Less accurate than SILVA	Very high accuracy and precision
**Input Data Required**	16S and 18S rRNA gene sequences<br>(FASTA format)	16S rRNA gene sequences<br>(FASTA format)	Metagenomic shotgun sequence data<br>(FASTA, FASTAq., tar.bz2)
**Output Data Type**	Relative abundance of OTUs<br>Taxonomic lineage<br>Phylogenetic trees	Relative abundance of OTUs<br>Taxonomic assignments<br>Phylogenetic trees	Relative abundance of taxonomic units (species or strains)<br>Phylogenetic analysis
**Strengths**	Wide taxonomic range<br>Quality-checked records.<br>User-friendly website interface	User-friendly interface	Able to identify and quantify species and strain in metagenomic data
**Limitations**	Misclassification can occur at the strain level, and there is an inability to identify novel strains.	Infrequent updates may lead to misclassification and the inability to identify novel bacterial sequences. Recently, in 2020, it updated with a more extensive repository of bacterial sequences.	Misidentify strains not covered in its database.<br>Requires multiple CPUs
**Reviewed study**	Peng et al (2020)	Pi et al (2020)<br>Cheng et al (2022)	Peng et al (2020)

Abbreviations:
MetaPhlAn2: Metagenomic Phylogenetic Analysis version 2; **16S rRNA**: small sub-units of ribosomal -RNA-ribonucleic acid; **ARB:** "arbor = tree"; software environment for rRNA sequence data; **NCBI:** national center for bioinformatics; **OTUs:** operational taxonomic units; **FASTA:** 'fast-all' means any text-based format; DNA or protein sequence; **FASTq**.: text-based format which include DNA sequence and its quality scores; **Tar.bz2**: tarball compressed to gzib files; **CPU:** Central Processing Unit

References:

1. Quast C, Pruesse E, Yilmaz P, Gerken J, Schweer T, Yarza P, et al. The SILVA ribosomal RNA gene database project: improved data processing and web-based tools. Nucleic Acids Res. 2013 Jan;41(Database issue):D590–6.

2. Ceccarani C, Severgnini M. A comparison between Greengenes, SILVA, RDP, and NCBI reference databases in four published microbiota datasets [Internet]. Bioinformatics; 2023 Apr [cited 2023 Dec 22]. Available from: http://biorxiv.org/lookup/doi/10.1101/2023.04.12.535864

3. Documentation [Internet]. [cited 2023 Dec 22]. Available from: https://www.arb-silva.de/documentation/

4. Balvočiūtė M, Huson DH. SILVA, RDP, Greengenes, NCBI and OTT — how do these taxonomies compare? BMC Genomics. 2017 Mar 14;18(2):114.

5. Park SC, Won S. Evaluation of 16S rRNA Databases for Taxonomic Assignments Using Mock Community. Genomics Inform. 2018 Dec;16(4):e24.

6. McDonald D, Jiang Y, Balaban M, Cantrell K, Zhu Q, Gonzalez A, et al. Greengenes2 unifies microbial data in a single reference tree. Nat Biotechnol. 2023 Jul 27;1–4.

7. QIIME 2 Forum [Internet]. 2023 [cited 2023 Dec 22]. Introducing Greengenes2 2022.10. Available from: https://forum.qiime2.org/t/introducing-greengenes2-2022-10/25291

8. https://mothur.org [Internet]. [cited 2023 Dec 22]. Greengenes-formatted databases. Available from: https://mothur.org

9. Blanco-Míguez A, Beghini F, Cumbo F, McIver LJ, Thompson KN, Zolfo M, et al. Extending and improving metagenomic taxonomic profiling with uncharacterized species using MetaPhlAn 4. Nat Biotechnol. 2023 Nov;41(11):1633–44.

10. Liu YX, Qin Y, Chen T, Lu M, Qian X, Guo X, et al. A practical guide to amplicon and metagenomic analysis of microbiome data. Protein Cell. 2021 May;12(5):315–30.

11. MetaPhlAn 2: Metagenomic Phylogenetic Analysis [Internet]. bioBakery; 2023 [cited 2023 Dec 22]. Available from: https://github.com/biobakery/MetaPhlAn2
