# Supplementary material for: Microbiota composition effect on immunotherapy outcomes in colorectal cancer patients: A systematic review
Source: PLoS One. 2024 Jul 24;19(7):e0307639. doi: 10.1371/journal.pone.0307639 (PMC11268651; doi:10.1371/journal.pone.0307639)
Supplement: S4 Table — (PDF) [file pone.0307639.s005.pdf]

Table S4. Overview of bioinformatics tools utilized for functional pathway prediction in the reviewed studies.

**Comparison Parameters**	**UniRef90 database**(1)	**DIAMOND tool**(2)	**Pangenome tool**(3,4)	**KEGG database**(5)	**MetaCyc database**(6)	**PICRUSt tool**(7,8)	**Bug Base tool**(9)	**HUMAnN2 pipeline**(10)
**Purpose**	Provides clustered sets of sequences from UniProt Knowledgebase and selected UniParc records	Sequence mapping tool, aligner for protein, and translated DNA searches.	A mapping tool is used to group sequences to identify the functional potential of taxa, species, and strains in a bacterial population.	It integrates genomic, chemical, and systemic functional information.	Validated experimentally metabolic pathways	Predicts microbial communities and functional profiling	Predict organism-level microbiome phenotypes	Determine the presence/absence and abundance of microbial pathways
**Database Size**	Large-scale clustering of the whole UniProt database	Able to handle huge databases and sequencing datasets.	Uses various public databases, mostly Interpro, KEGG, COG, Pfam	Extremely large, it contains data from more than 20,690 organisms	Includes thousands of pathways from over 2800 organisms.	Uses the KEGG database	Reference dataset of 3605 bacterial and archaeal genomes	Uses the UniRef and ChocoPhlAn databases
**Required Input Data**	Protein sequences (including Isoforms)	DNA and protein sequences.<br>FASTA, FASTAq,	Genes or protein sequences;<br>FASTA, FASTAq	Gene sequences, protein sequences and chemical structures	Genomic or metagenomic data.	16S rRNA gene sequences	OTU tables of 16S rRNA gene sequences	Metagenomic or metatranscriptomic sequencing reads
**Generated Output Data**	A clustered output of the protein sequences.	Alignment files in various formats.	Pangenome graph .gfa files, which are further converted to VCF files for further process	Biochemical pathways and interacting networks.<br>Gene Ontology terms	Metabolic pathway information.	Predicted metagenome function	Predicted microbiome phenotypes	Presence, absence, and abundance of microbial pathways
**Level of Taxonomic Detail**	Predicts functions down to strain level	Group the genetic composition down to the genus/species level	Group the genetic composition down to the strains level	Prediction down to species level.	Prediction down to species level	Prediction down to genus level	Prediction down to the species level	Prediction down to the strain level
**Functions Predicted**	Provides sequence space, functional annotation, and taxonomic diversity.	Predicts functionality based on reference database	Functional distribution in a community	Metabolic pathways, information processing of genetic, cellular, environmental data, organismal systems, and human diseases	Predicts a comprehensive range of metabolic pathways.	Metabolic pathways	Phenotypic characterizations	Metabolic reconstruction at both pathway and reaction level
**Strengths and Ease of use**	Reduces redundancy and improves speed of sequence similarity searches<br><br>Convenient for large-scale computational analysis	High speed; ability to process and match large databases rapidly.	Designed for integration with metagenomic data, which helps overcome inherent analytical limitations<br><br>Allow comparative analysis across microbial communities.	Provides highly detailed and broad-range pathways.	Extensive information on metabolic pathways across numerous organisms.<br><br>User-friendly with well-annotated and curated data.	Allows representation of entire microbial communities<br><br>Relatively user-friendly with a good range of online tutorials	Can provide a functional understanding of a microbial community without needing sequence data	Can identify novel pathways and microbes; provides strain-level resolution
**Limitations**	Limited taxonomic prediction.	Accuracy depends on the reference database used.<br><br>Requires advanced technical knowledge	Due to the limited metagenomic coverage, it is still a challenging task.<br>Efforts need to be made to improve the accuracy of the assembly	Not efficient at strain level prediction.<br><br>Requires a high level of bioinformatics understanding and may have a steep learning curve	Primarily focused on metabolic pathways, it may lack some broader functional predictions.	Cannot predict strain-level differences or deal with unknown genes	Limited by the quality and depth of the OTUs, limited to bacteria and archaea<br><br>Requires bioinformatics skills and specific software	Computationally intensive; requires high-quality data.<br><br>User-friendly but requires computational skills.
**Reviewed Study**	Peng et al., (2020)	Peng et al., (2020)	Peng et al., (2020)	Peng et al., (2020)<br>Pi et al., (2020)	Peng et al., (2020)	Pi et al., (2020)<br>Cheng et al., (2022)	Cheng et al., (2022)	Peng et al., (2020)

UniRef90: UniProt Reference Clusters with a 90% sequence identity in the clusters; **UniProt:** Universal Protein resource; **UniParc:** Universal Protein Archive; **KEGG:** Kyoto Encyclopedia of Genes and Genomes; **MetaCyc:** Metabolic Cyclical database; **PICRUSt:** Phylogenetic Investigation of Communities by Reconstruction of Unobserved States; **HUMAnN2:** Human Microbiome Project Unified Metabolic Analysis Network**; 16SrRNA:** small sub-units of ribosomal -RNA-ribonucleic acid; **DNA:** deoxynucleic acid**; COG:** clusters of orthologous genes; **Pfam:** protein families database; **InterPro:** Inter (integrated or interactive) and Pro (proteins); **FASTA:** ‘fast-all’ means any text-based format; DNA or protein sequence; **FASTq.:** text-based format which include DNA sequence and its quality scores; **OTUs:** operational taxonomic units; **VCF:** virtual contact files

References:

1. UniRef | UniProt help | UniProt [Internet]. [cited 2023 Dec 25]. Available from: https://www.uniprot.org/help/uniref

2. Buchfink B, Reuter K, Drost HG. Sensitive protein alignments at tree-of-life scale using DIAMOND. Nat Methods. 2021 Apr;18(4):366–8.

3. Rouli L, Merhej V, Fournier PE, Raoult D. The bacterial pangenome as a new tool for analysing pathogenic bacteria. New Microbes and New Infections. 2015 Sep 1;7:72–85.

4. Zhong C, Chen C, Wang L, Ning K. Integrating pan-genome with metagenome for microbial community profiling. Computational and Structural Biotechnology Journal. 2021 Jan 1;19:1458–66.

5. KEGG Release Notes [Internet]. [cited 2023 Dec 22]. Available from: https://www.genome.jp/kegg/docs/relnote.html

6. MetaCyc: Metabolic Pathways From all Domains of Life [Internet]. [cited 2023 Dec 22]. Available from: https://metacyc.org/

7. GitHub [Internet]. [cited 2023 Dec 22]. Home. Available from: https://github.com/picrust/picrust2/wiki/Home

8. Douglas GM, Maffei VJ, Zaneveld JR, Yurgel SN, Brown JR, Taylor CM, et al. PICRUSt2 for prediction of metagenome functions. Nat Biotechnol. 2020 Jun;38(6):685–8.

9. Ward T, Larson J, Meulemans J, Hillmann B, Lynch J, Sidiropoulos D, et al. BugBase predicts organism-level microbiome phenotypes [Internet]. bioRxiv; 2017 [cited 2023 Dec 22]. p. 133462. Available from: https://www.biorxiv.org/content/10.1101/133462v1

10. ATTENTION [Internet]. bioBakery; 2023 [cited 2023 Dec 22]. Available from: https://github.com/biobakery/humann
