# Supplementary material for: Microbiota composition effect on immunotherapy outcomes in colorectal cancer patients: A systematic review
Source: PLoS One. 2024 Jul 24;19(7):e0307639. doi: 10.1371/journal.pone.0307639 (PMC11268651; doi:10.1371/journal.pone.0307639)
Supplement: S5 Table — (PDF) [file pone.0307639.s006.pdf]

Table S5. Comparison of identified microbiota taxa in responders vs. non-responders across different tumor sites, treatment regimens, and time points in the reviewed studies

Reviewed study	Colorectal cancer		Gastric cancer		Esophageal Cancer	
	Responder group	Non-responder group	Responder group	Non-responder group	Responder group	Non-responder group
**Peng et al., 2020**<br>**Differential enrichment' findings**	TU104:<br>Genus: *Lachnoclostridium*<br>OTU294:<br>Genus: *Parabacteroides*<br>OTU293:<br>Genus: *Parabacteroides*<br>OTU155:<br>Genus: *Lachnospira*<br>OTU187:<br>Family: *Ruminococcaceae*<br>OTU197:<br>Genus: *Flavonifractor*<br>OTU18:<br>Genus: *Dialister*<br>OTU190:<br>Family: *Ruminococcaceae*<br>OTU77:<br>Family: *Lachnospiraceae*<br>OTU103:<br>Family: *Lachnospiraceae*<br>OTU271:<br>Genus: *Bacteroides*<br>OTU221:<br>Genus: *Ruminococcus*_2<br>OTU188:<br>Family: *Ruminococcaceae*<br>Species:<br>*Eubacterium rectale*<br>Species:<br>*Akkermansia muciniphila*	OTU274:<br>Genus: *Bacteroides*<br>OTU319:<br>Genus: *Odoribacter*<br>OTU176:<br>Genus: *Oscillibacter*<br>OTU201:<br>Genus: *Bifidobacterium*<br>OTU52:<br>Genus: *Lachnoclostridium*<br>OTU61:<br>Genus: *Subdoligranulum*<br>OTU57:<br>Genus: *Subdoligranulum*<br>OTU228:<br>Genus: *Coprococcus*_2<br>OTU304:<br>Genus: *Parabacteroides*<br>OTU282:<br>Genus: *Bacteroides*	OTU263:<br>Genus: *Prevotella*<br>Species: *Prevotella*_9<br>OTU202:<br>Genus: *Bifidobacterium*<br>OTU264:<br>Genus: *Prevotella*<br>Species: *Prevotella*_2<br>OTU156:<br>Genus: *Lachnospira*<br>OTU274:<br>Genus: *Bacteroides*<br>OTU190:<br>Family: *Ruminococcaceae*<br>OTU79:<br>Genus: *Agathobacter*<br>OTU275:<br>Genus: *Bacteroides*	OTU281:<br>Genus: *Bacteroides*<br>OTU147:<br>Genus: *Agathobacter*<br>OTU201:<br>Genus: *Bifidobacterium*<br>OTU76:<br>Family: *Lachnospiraceae*<br>OTU312:<br>Genus: *Butyricimonas*<br>OTU25:<br>Genus: *Megamonas*	OTU261:<br>Genus: *Prevotella*<br>Species: *Prevotella*_9<br>OTU18:<br>Genus: *Dialister*<br>OTU97:<br>Family: *Lachnospiraceae*<br>OTU222:<br>Genus: *Ruminococcus*_2<br>OTU266:<br>Genus: *Bacteroides*<br>OTU207:<br>Genus: *Parasutterella*<br>OTU1:<br>Genus: *Phascolarctobacterium*<br>OTU277:<br>Genus: *Bacteroides*<br>OTU105:<br>Family: *Lachnospiraceae*<br>OTU128:<br>Family: *Lachnospiraceae*<br>OTU308:<br>Genus: *Paraprevotella*	OTU158:<br>Family: *Lachnospiraceae*<br>OTU153:<br>Family: *Lachnospiraceae*<br>OTU172:<br>Family: *Ruminococcaceae*<br>OTU183:<br>Family: *Ruminococcaceae*<br>OTU274:<br>Genus: *Bacteroides*<br>OTU113:<br>Genus: *Marvinbryantia*<br>OTU124:<br>Genus: *Hungatella*<br>OTU131:<br>Genus: *Clostridium*_sensu<br>OTU35:<br>Genus: *Lactobacillus*<br>OTU27:<br>Genus: *Bacteroides*
All patients were treated anti–PD-1/PD-L1 immune-checkpoint inhibitors; Nivolumab ± Ipilimumab.						

Table S5. **Comparison of identified microbiota taxa in responders vs. non-responders across different tumor sites, treatment regimens, and time points in the reviewed studies**

Reviewed study	Colorectal cancer treated with PD-1 monoclonal antibody therapy*			Colorectal cancer treated with chemotherapy			
	Responder group		Non-responder group	Responder group		Non-responder group	
**Pi et al., 2020**<br>**Abundance-based findings**	CAG00646:<br>Genus: Alistipes<br>Species: Not specified (1668)<br>CAG00301:<br>Genus: Akkermansia<br>Species: muciniphila (3187)<br>CAG00363:<br>Genus: Intestinimonas<br>Species: Not specified (2056)<br>CAG00064:<br>Unclassified at the specified taxonomic level (3310)<br>CAG00116:<br>Genus: Bacteroides<br>Species: nordi (2783)<br>CAG01090:<br>Unclassified Firmicutes (1026)<br>CAG00945:<br>Genus: Bacteroides<br>Species: xylanisolvens (2321)<br>CAG01262:<br>Genus: Blautia<br>Species: Not specified (750)<br>CAG01227:<br>Unclassified at the specified taxonomic level (815)<br>CAG00871:<br>Family: Lachnospiraceae<br>Genus: Not specified (1362)<br>CAG00510:<br>Genus: Alistipes<br>Species: Not specified (1860)<br>CAG00670:<br>Unclassified Firmicutes (1648)<br>CAG00862:<br>Genus: Firmicutes bacterium CAG:129<br>Species: Not specified (1390)<br>CAG01245:<br>Phylum: Firmicutes<br>Class: Not specified (780)<br>CAG00676:<br>Unclassified Firmicutes (1643)<br>CAG:353<br>Species: Not specified (2262)	CAG00604:<br>Genus: Firmicutes bacterium CAG:110<br>Species: Not specified (1714)<br>CAG00559:<br>Unclassified Clostridiales (1776)<br>CAG01200:<br>Order: Clostridiales<br>Family: Not specified (863)<br>CAG00913:<br>Unclassified at the specified taxonomic level (1291)<br>CAG00854:<br>Family: Ruminococcaceae<br>Genus: Not specified (2572)<br>CAG00629:<br>Genus: Firmicutes bacterium CAG:124<br>Species: Not specified (1684)<br>CAG00317:<br>Genus: Clostridium sp. CAG:230<br>Species: Not specified (2130)<br>CAG00555:<br>Genus: Flavonifractor<br>Species: Not specified (1782)<br>CAG00391:<br>Unclassified Clostridiales (2019)<br>CAG00049:<br>Genus: Bacteroides<br>Species: caceae<br>Species: Not specified (3561)<br>CAG00695:<br>Unclassified Firmicutes (1618)<br>CAG00994:<br>Unclassified Firmicutes (1171)<br>CAG01342:<br>Family: Ruminococcaceae<br>Genus: Unclassified (613)<br>CAG00250:<br>Genus: Ruminococcus sp.	CAG00690:<br>Order: Clostridiales<br>Family: Unclassified Clostridiales<br>Genus: Unclassified<br>Species: Not specified (1629)<br>CAG00141:<br>Genus: Parabacteroides<br>Species: distasonis (2649)<br>CAG00211:<br>Phylum: Firmicutes<br>Genus: Firmicutes bacterium CAG:227<br>Species: Not specified (2389)<br>CAG00048_1:<br>Order: Clostridiales<br>Species: Not specified (1403)<br>CAG00168:<br>Order: Clostridiales<br>Genus: Clostridiales VE202-14<br>Species: Not specified (2534)<br>CAG00720:<br>Genus: Anaerotruncus<br>Species: colhominis (1590)<br>CAG01401:<br>Family: Lachnospiraceae<br>Species: Not specified (522)	CAG00469:<br>Genus: Eubacterium<br>Species: Not specified (1928)<br>Strain: CAG:146<br>CAG00871:<br>Family: Lachnospiraceae<br>Genus: Not specified (1362)<br>CAG01090:<br>Phylum: Firmicutes<br>Class: Not specified<br>Order: Not specified<br>Family: Not specified<br>Genus: Unclassified Firmicutes<br>Species: Not specified (1026)<br>Erysipelotrichaceae bacterium 5_2_54FAA:<br>Family: Erysipelotrichaceae<br>Genus: Bacterium<br>Species: 5_2_54FAA<br>CAG00134:<br>Genus: Cloacibacillus<br>Species: porcorum (2690)<br>CAG00347:<br>Genus: Enterococcus<br>Species: faecium (2087)<br>CAG00363:<br>Genus: Intestinimonas<br>Species: Not specified (2056)<br>CAG01227:<br>Unclassified at the specified taxonomic level (815)<br>CAG00821:	Order: Clostridiales<br>Genus: Unclassified<br>Species: Not specified (1461)<br>CAG00646:<br>Genus: Alistipes<br>Species: Not specified (1668)<br>CAG01223:<br>Unclassified Firmicutes (819)<br>CAG00355:<br>Genus: Bacteroides<br>Species: Not specified<br>Strain: CAG:661 (2067)<br>CAG00328:<br>Genus: Alistipes<br>Species: indistinctus (2113)<br>CAG00966:<br>Genus: Firmicutes bacterium CAG:552<br>Species: Not specified (1209)<br>CAG00892:<br>Genus: Firmicutes<br>Species: Not specified (1324)<br>CAG00530:<br>Genus: Prevotella<br>Species: Not specified (1821)<br>CAG00391:<br>Unclassified Clostridiales (2019)<br>CAG00676:<br>Unclassified Firmicutes (1643)<br>CAG01308:<br>Unclassified Firmicutes (666)	CAG01112:<br>Unclassified<br>Species: Not specified (1003)<br>CAG01004:<br>Genus: Prevotella<br>Species: Not specified (1161)<br>CAG00960:<br>Genus: Clostridium<br>Species: sp. CAG:921 (1213)<br>CAG00658:<br>Phylum: Firmicutes<br>Class: Not specified<br>Order: Not specified<br>Family: Not specified<br>Genus: Unclassified Firmicutes<br>Species: Not specified (1661)<br>CAG00473:<br>Genus: Prevotella<br>Species: sp. CAG:617 (1920)<br>CAG00308:<br>Unclassified (2153)<br>CAG01161:<br>Unclassified (934)<br>CAG00137:<br>Order: Clostridiales<br>Genus: Unclassified<br>Species: Not specified (2673)<br>CAG00048_1:<br>Order: Clostridiales<br>Species: Not specified (1403)	CAG00008:<br>Genus: Clostridium<br>Species: bolteae (6646)<br>CAG00211:<br>Phylum: Firmicutes<br>Genus: Firmicutes bacterium CAG:227<br>Species: Not specified (2389)<br>CAG00168:<br>Order: Clostridiales<br>Genus: Clostridiales bacterium VE202-14<br>Species: Not specified (2534)<br>CAG01214:<br>Genus: Blautia<br>Species: Not specified (832)<br>CAG00175:<br>Genus: Bacteroides<br>Species: clarus (2509)<br>CAG00835:<br>Phylum: Proteobacteria<br>Genus: Unclassified<br>Species: Not specified (1444)<br>CAG00065:<br>Genus: Blautia<br>Species: Not specified (3272)<br>CAG00116:<br>Genus: Bacteroides<br>Species: nordi (2783)
*Pembrolizumab							

Table 5. Comparison of identified microbiota taxa in responders vs. non-responders across different tumor sites, treatment regimens, and time points in the reviewed studies

**Reviewed study**	**Advanced Cancer***			
	**Response group before immunotherapy**	**Response group after immunotherapy**	**Non-response group before immunotherapy**	**Non-response group after immunotherapy**
**Cheng et al., 2022**<br>**Abundance-based findings**	R.0:<br><br>**Phylum:**<br>*Firmicutes*<br>*Bacteroidetes*<br>*Proteobacteria*<br>*Actinobacteria*<br><br>**Family:**<br>Bacteroidaceae (22.55–25.26%)<br>*Lachnospiraceae* (12.51–15.68%)<br>*Ruminococcaceae* (11.89–16.01%)<br><br>**Genus:**<br>*Bacteroides* (22.55–25.26%)<br>*Prevotella* (7.44–12.03%)<br>*Faecalibacterium* (5.01%)<br>*Megamonas* (3.46%)	R.T:<br><br>**Phylum:**<br>*Firmicutes*<br>*Bacteroidetes*<br>*Proteobacteria*<br>*Actinobacteria*<br><br>**Family:**<br>Bacteroidaceae (22.55–25.26%)<br>Lachnospiraceae (12.51–15.68%)<br>Ruminococcaceae (11.89–16.01%)<br><br>**Genus:**<br>*Bacteroides* (22.55–25.26%)<br>*Prevotella* (7.44–12.03%)<br>*Faecalibacterium* (4.29%)<br>*Bifidobacterium* (2.66%)	NR.0:<br><br>**Phylum:**<br>*Firmicutes*<br>*Bacteroidetes*<br>*Proteobacteria*<br>*Actinobacteria*<br><br>**Family:**<br>*Bacteroidaceae* (22.55–25.26%)<br>*Lachnospiraceae* (12.51–15.68%)<br>*Ruminococcaceae* (11.89–16.01%)<br>*Prevotellaceae* (10.90–12.03%)<br><br>**Genus:**<br>*Bacteroides* (22.55–25.26%)<br>*Prevotella* (7.44–12.03%)<br>*Faecalibacterium* (4.78%)<br>*Bifidobacterium* (3.67%)	NR.T:<br><br>**Phylum:**<br>*Firmicutes*<br>*Bacteroidetes*<br>*Proteobacteria*<br>*Actinobacteria*<br><br>**Family:**<br>*Bacteroidaceae* (22.55–25.26%)<br>*Lachnospiraceae* (12.51–15.68%)<br>*Ruminococcaceae* (11.89–16.01%)<br>*Enterobacteriaceae* (11.50%)<br>*Veillonellaceae* (9.65%)<br><br>**Genus:**<br>*Bacteroides* (22.55–25.26%)<br>*Prevotella* (7.44–12.03%)<br>*Faecalibacterium* (4.78%)<br>*Bifidobacterium* (3.67%)<br>*Veillonella* (3.43%)<br>*Lachnospira* (2.98%)

*Non-squamous non-small cell lung cancer; Lung squamous cell carcinoma; Hepatocellular carcinoma; Gastric cancer; Colorectal carcinoma; Melanoma; Nasopharyngeal carcinoma; Cervical cancer; Small-cell lung cancer; laryngeal cancer; osteosarcoma; renal pelvic carcinoma; bladder cancer; pancreatic cancer; esophageal cancer; ureteral cancer; mediastinal carcinoma; and cholangiocarcinoma

PD-1 pathway inhibitors used in the study: Nivolumab; Pembrolizumab; Sintilimab; Camrelizumab; and Toripalimab

Table S5. Comparison of identified microbiota taxa in responders vs. non-responders across different tumor sites, treatment regimens, and time points in the reviewed studies

**Reviewed study**	**Advanced Cancer***			
	**Response group before immunotherapy**	**Response group after immunotherapy**	**Non-response group before immunotherapy**	**Non-response group after immunotherapy**
**Cheng et al., 2022**<br>**Differential enrichment' findings**	**Enriched in R.0:**<br><br>**Domain:**<br>Archaea<br><br>**Phylum:**<br>*Lentisphaerae*<br>*Euryarchaeota*<br><br>**Class:**<br>*Lentisphaeria*<br>*Methanobacteria*<br><br>**Order:**<br>*Victivallales*<br>*Methanobacteriales*<br><br>**Family:**<br>*Victivallaceae*<br>*Methanobacteriaceae*<br><br>**Genus:**<br>*Methanobrevibacter*<br>*Leuconostoc*	**Enriched in R.T**<br><br>**Family:**<br>*Lachnospiraceae*<br><br>**Genus:**<br>*Thermus*	**Enriched in NR.0:**<br><br>**Family:**<br>*Clostridiaceae*	NM
**ROC Analysis (Diagnostic Value)** ‡	**R.0: AUC = 0.65**	**R.T:** AUC = 0.70	**NR.0:** AUC = 0.62	**NR.T:** AUC = 0.64

*Non-squamous non-small cell lung cancer; Lung squamous cell carcinoma; Hepatocellular carcinoma; Gastric cancer; Colorectal carcinoma; Melanoma; Nasopharyngeal carcinoma; Cervical cancer; Small-cell lung cancer; laryngeal cancer; osteosarcoma; renal pelvic carcinoma; bladder cancer; pancreatic cancer; esophageal cancer; ureteral cancer; mediastinal carcinoma; and cholangiocarcinoma

‡ The ROC analysis suggests that the microbiota has diagnostic value in differentiating the four groups of cancer patients, with varying AUC (Area under the curve) values for each group.

NM: Not mentioned

PD-1 pathway inhibitors used in the study: Nivolumab; Pembrolizumab; Sintilimab; Camrelizumab; and Toripalimab

Table S5. Comparison of identified microbiota taxa in responders vs. non-responders across different tumor sites, treatment regimens, and time points in the reviewed studies

<table>
<tr><th rowspan="2">Reviewed study</th><th colspan="4">Advanced Cancer*</th></tr>
<tr><th>Response group before immunotherapy</th><th>Response group after immunotherapy</th><th>Non-response group before immunotherapy</th><th>Non-response group after immunotherapy</th></tr>
<tr><td>Cheng et al., 2022
Abundance-based findings</td><td colspan="4">Top 10 microbial composition at the phylum level and genus level across the 4 groups (R.0, R.T, NR.0, NR.T)

Top phyla
• Firmicutes
• Bacteriodetes
• Lachnospiraceae
• Ruminococceae
• Verrucomicrobia
• Fusobacteria
• Synergistetes
• Tenericutes
• Lentisphaerae
• TM7

Top genera
• Bacteroides
• Prevotella
• Faecalibacterium megamonas
• Bifidobacterium
• Veillonell
• Lachnospira
• Phascolarctobacterium
• Ruminicoccus
• Parabacteroides</td></tr>
</table>

*Non-squamous non-small cell lung cancer; Lung squamous cell carcinoma; Hepatocellular carcinoma; Gastric cancer; Colorectal carcinoma; Melanoma; Nasopharyngeal carcinoma; Cervical cancer; Small-cell lung cancer; laryngeal cancer; osteosarcoma; renal pelvic carcinoma; bladder cancer; pancreatic cancer; esophageal cancer; ureteral cancer; mediastinal carcinoma; and cholangiocarcinoma
.
PD-1 pathway inhibitors used in the study: Nivolumab; Pembrolizumab; Sintilimab; Camrelizumab; and Toripalimab

Table S5. Comparison of identified microbiota taxa in responders vs. non-responders across different tumor sites, treatment regimens, and time points in the reviewed studies

Reviewed study	Colorectal Cancer*	
	**Response group**	**Non-response group**
**Koptez et al., 2017**<br>**Differential enrichment' findings**	**Differentially depleted:**<br><br>**Family:**<br>*Micrococcaceae*<br>**Genus:**<br>*Rothia*<br>**Species:**<br>*mucilaginosa*	**Differentially enriched:**<br><br>**Family:**<br>*Micrococcaceae*<br>**Genus:**<br>*Rothia*<br>**Species:**<br>*mucilaginosa*

PD-1 pathway inhibitors used in the study: Nivolumab ± Ipilimumab
