# Supplementary material for: Microbiota composition effect on immunotherapy outcomes in colorectal cancer patients: A systematic review
Source: PLoS One. 2024 Jul 24;19(7):e0307639. doi: 10.1371/journal.pone.0307639 (PMC11268651; doi:10.1371/journal.pone.0307639)
Supplement: S6 Table — (PDF) [file pone.0307639.s007.pdf]

Table S6. Microbiota taxa utilized in the phylogenetic tree construction in Fig 2

Reviewed study	Responder group	Non-responder group
**a.** Peng et al.,2020	OTU77|Lachnospiraceae<br>OTU103|Lachnospiraceae<br><br>OTU155|Lachnospira<br><br>OTU271|Bacteroides<br>OTU104|Lachnoclostridium<br>OTU294|Parabacteroides<br>OTU293|Parabacteroides<br>OTU197|Flavonifractor<br>OTU18|Dialister<br>Eubacterium rectale<br><br>OTU221|Ruminococcus_2<br>OTU188|Ruminococcaceae_UCG-005<br>OTU187|Ruminococcaceae_UCG-005<br>OTU190|Ruminococcaceae_UCG-005<br>OTU103|Lachnospiraceae_AC2044 *	OTU274|Bacteroids<br>OTU282|Bacteroides<br><br>OTU52| Lachnoclostridium<br>OTU319|Odoribactr<br>OTU176|Oscillibacr<br>OTU201|Bifidobacterium<br>OTU61|Subdoligranulum<br>OTU57|Subdoligranulum<br>OTU228|Coprococcus_2<br>OTU304|Parabacteroides
**b.** Pi et al.,2020	CAG01245, 780|Firmicutes<br><br>CAG00871, 1362| Lachnospiraceae<br>CAG00854, 2572| Ruminococcaceae<br><br>CAG01200, 863| Clostridiales<br><br>CAG00555, 1782| Flavonifractor<br>CAG00646, 1668| Alistipes<br>CAG00510, 1860| Alistipes<br>CAG01262, 750| Blautia<br>CAG00363, 2056| Intestinimonas<br>CAG00116, 2783| Bacteroides nordii<br>CAG00049, 3561| Bacteroides caccae<br>CAG00945, 2321| Bacteroides xylanisolvens<br>CAG00301, 3187|Akkermansia muciniphila<br>CAG00862, 1390| Firmicutes bacterium CAG:129<br>CAG00604, 1714| Firmicutes bacterium CAG:110<br>CAG00629, 1684| Firmicutes bacterium CAG:124<br>CAG00250, 2262| Ruminococcus sp. CAG:353<br>CAG00317, 2130| Clostridium sp. CAG:230<br>CAG00695, 1618| unclassified Firmicutes<br>CAG00994, 1171| unclassified Firmicutes<br>CAG00670, 1648| unclassified Firmicutes<br>CAG00676, 1643| unclassified Firmicutes<br>CAG01342, 613| unclassified Ruminococcaceae<br>CAG00391, 2019| unclassified Clostridiales<br>CAG00559, 1776| unclassified Clostridiales<br>CAG00064, 3310| unclassified<br>CAG01227, 815| unclassified<br>CAG00913, 1291| unclassified	C. G000 8_1, 1403|Clostridiales<br><br>AG01401, 522| Lachnospiraceae<br><br>CAG00141, 2649| Parabacteroides distasonis<br>G00720, 1590| Anaerotruncus colihominis<br>C Go 211, 2389| Firmicutes bacterium CAG:227<br>AL 10168, 2534| Clostridiales VE202-14<br>CAG00690, 1629| unclassified Clostridiales
**c.** Kopetz et al., 2017	NM	Micrococcaceae ***<br>Rothia<br>Rothia Mucilaginosa

*Renamed taxa in the tree: some taxa names were not recognized by the software used to construct the phylogenetic tree, and therefore needed to be renamed for the process.
*** Manually added families to the phylogenetic tree
NM: Not mentioned
