# Supplementary material for: Microbiota composition effect on immunotherapy outcomes in colorectal cancer patients: A systematic review
Source: PLoS One. 2024 Jul 24;19(7):e0307639. doi: 10.1371/journal.pone.0307639 (PMC11268651; doi:10.1371/journal.pone.0307639)
Supplement: Attachment — Submitted filename: Review comments.docx [file pone.0307639.s008.docx]

Dear Editor,

Thanks for the opportunity to review the manuscript titled " Microbiota composition effect on immunotherapy outcomes in colorectal cancer patients: a systematic review ".

The manuscript investigates whether composition of gut microbiota could potentially serve as a biomarker for predicting the clinical effectiveness of immune checkpoint inhibitors in CRC patients. Phyla including Firmicutes, Bacteroidata, Actinobacteria, and Verrucomicrobiota were associated with CRC patients’ clinical response toward ICIs treatment. Ruminococcaceae and few more families of gut bacteria were found to be differentially related to clinical response of CRC patients. This research concludes that there is need for additional comprehensive, multicentre studies with larger sample sizes to validate reported microbiota and expand the understanding of the role of gut microbiota in CRC for ICIs therapy. The study's design incorporates suitable method to achieve the outlined objective. The content is logically structured, providing cohesive information.

The manuscript can be considered for publication. However, the following points need to be addressed -

**From Methods Section:**

1. Authors could elaborate the rationale behind the decision to include only published observational studies and exclude experimental animal studies, reviews, comments, case reports, editorials, and conference abstracts?

2. Authors could elaborate on the methodologies used by some most sited studies to analyse the differential observations regarding gut microbiota composition of colorectal cancer patients undergoing immunotherapy?

3. Were there any notable differences in patient recruitment strategies among the included studies, and how might these differences impact the generalizability of their findings?

4. The article could provide a comprehensive overview of the role of phyla based gut microbiota effects in colorectal cancer and its response to immunotherapy.

**From Results Section:**

1. Can you elaborate on the characteristics of the studies included in the analysis, such as their sample sizes, publication years, and geographic locations?

2. How did the studies by Cheng *et al.,* Peng *et al.,* and Kopetz *et al.* differ in their methodologies and findings regarding the relationship between gut microbiome composition and response to immunotherapy in colorectal cancer patients (except the clinical trials)?

3. What were the primary outcomes regarding the predictive taxonomic profiles of colorectal cancer patients in response to immune checkpoint inhibitors (ICIs) based on the included studies?

**Regarding the Bias Section**

1. Can the authors explain the potential sources of bias identified in the included studies, such as methodological and reporting limitations?

2. How did the authors address or mitigate the impact of these biases on the internal validity of the systematic review?

**Regarding strength and limitations**

1. What were the main strengths of the reviewed studies, and how do they contribute to the understanding of the connection between gut microbiota and immune checkpoint inhibitors (ICIs)?

2. How might the limitations of the included studies, such as small sample sizes and geographic bias, impact the generalizability of the findings and the ability to draw comprehensive conclusions?

3. How did the studies address potential limitations in data interpretation, such as the dynamic nature of the microbiome and the variability in microbial composition among individuals?

**Regarding future clinical relevance**

1. Based on the findings of the included studies, what implications do these results have for the development of personalized treatment strategies for colorectal cancer patients receiving immunotherapy?

2. Were there any consistent patterns or trends observed across the studies that could inform clinical decision-making regarding the use of immunotherapy in colorectal cancer patients with specific microbiome profiles?

3. How might the identification of predictive taxonomic profiles for immunotherapy response in colorectal cancer patients impact future research and clinical practice in oncology?

4. Are there specific aspects of the gut microbiome or immunotherapy response that were not adequately addressed by the included studies, and if so, what areas warrant further investigation?

5. How might advances in technology and methodology, such as multi-omics approaches or longitudinal studies, enhance our understanding of the complex interplay between the microbiome and cancer immunotherapy outcomes?
