# Supplementary material for: Microbiota composition effect on immunotherapy outcomes in colorectal cancer patients: A systematic review
Source: PLoS One. 2024 Jul 24;19(7):e0307639. doi: 10.1371/journal.pone.0307639 (PMC11268651; doi:10.1371/journal.pone.0307639)
Supplement: Attachment — Submitted filename: PLOS ONE_Response to reviewers 05_06_2024_ZR.docx [file pone.0307639.s009.docx]

June 05, 2024

Vinod Kumar Yata
Academic Editor
PLOS ONE

***Ref PONE-D-24-10055: Microbiota composition effect on immunotherapy outcomes in colorectal cancer patients: a systematic review***

Dear Dr. Vinod Kumar Yata

We appreciate the feedback from the reviewers and the opportunity to submit a revised version of our manuscript. Following the comments provided, we have thoroughly revised the manuscript to incorporate the suggested amendments. For ease of review, the revised manuscript was displayed in track mode, and some paragraphs were highlighted, addressing some of the reviewer comments. Below, detailed responses to each of the reviewers’ comments are provided.

**Journal Requirements**

**Comment 1: Please ensure that your manuscript meets PLOS ONE’s style requirements, including those for file naming.**

**Answer:**

We confirm that our manuscript adheres to the style guidelines of PLOS ONE.

**Comment 2:** **We note that Figure 3 in your submission contains copyrighted images. All PLOS content is published under the Creative Commons Attribution License (CC BY 4.0), which means that the manuscript, images, and Supporting Information files will be freely available online, and any third party is permitted to access, download, copy, distribute, and use these materials in any way, even commercially, with proper attribution. We require you to either (1) present written permission from the copyright holder to publish these figures specifically under the CC BY 4.0 license, or (2) remove the figures from your submission:**

**Answer:**

The images in the manuscript were reproduced through the utilization of mindthegraph.com for Figure 3 and PowerPoint of Figure S1 in the format of PNG. Subsequently, a CC BY 4.0 license was acquired in response to a specific request.

**Reviewers’ comments**

**From Methods Section:**

**Comment 1: Authors could elaborate on the rationale behind the decision to include only published observational studies and exclude experimental animal studies, reviews, comments, case reports, editorials, and conference abstracts.**

**Answer:**

Thank you for your comment. As stated on Page 5, Paragraph 3 of our manuscript, the intent is to shed light on the association between gut microbiota and the clinical efficacy of immune checkpoint inhibitors in colorectal cancer patients, thus directly contributing to the clinical oncology community’s body of knowledge. Our research aims to provide a comprehensive overview of the existing understanding regarding the specified objective, with the intention of enhancing the methodologies employed by healthcare practitioners and researchers, consequently leading to favorable advancements in therapeutic approaches within cancer therapy.

To ensure the focus remained on applicable, human-centric data, we exclusively considered peer-reviewed published observational studies animal experiments were omitted from consideration due to the potential limitations in directly extrapolating findings to human subjects. Similarly, reviews, comments, case reports, editorials, and conference abstracts were not included; while these can offer valuable insights, they do not constitute the original research needed for our analysis. Reviews and comments typically summarize other findings, case reports provide anecdotal evidence with limited generalizability, editorials represent opinions, and conference abstracts may present unvetted preliminary data, apart from one abstract was included which was part of the international CheckMate 142 clinical trial. Our stringent selection criteria were applied to construct an analysis founded upon the most reliable and broadly applicable evidence.

Please refer to the revised Methods section on Page 7 for details on the amendments made.

**Comment 2: Authors could elaborate on the methodologies used by some most cited studies to analyze the differential observations regarding gut microbiota composition of colorectal cancer patients undergoing immunotherapy.**

**Answer:**

Thank you for your valuable comment. We recognize the importance of the methodologies used in the primary studies for analyzing gut microbiota composition among colorectal cancer patients undergoing immunotherapy. As part of our systematic review criteria, we stipulated that gut microbiota composition must be reported via molecular analysis, which we have detailed in the Methods section (page 6 Line 127).

Furthermore, we dedicated a section in our Results titled “Comparative Analysis of Methodologies Utilized in Microbiota Profiling,” (Page 21) wherein we critically assess the methodologies employed by the studies included in our review. This section is supported by relevant table S3,S4 and Figure S1 which systematically showcase the fundamental comparisons and contrasts between the reported methods in each paper. This includes the molecular techniques utilized for the profiling of microbiota, and the bioinformatic tools applied for analysis.

We believe this approach provides a comprehensive overview of the methodologies and allows readers to understand the potential impact of these methods on the study outcomes. However, if additional elaboration on specific methodologies used by the most cited papers is required, we are willing to provide further details and discussion as per the journal’s and the reviewers’ recommendations.

**Comment 3: Were there any notable differences in patient recruitment strategies among the included studies, and how might these differences impact the generalizability of their findings?**

**Answer:**

Thank you for bringing attention to the recruitment strategies of the included studies. As detailed in **Table.1** and the “Study Characteristics” section (page 14), we meticulously cataloged the inclusion and exclusion criteria for each study. Moreover, in the “Assessment of Risk of Bias” section (page 23), we discussed methodological and reporting limitations within the individual studies, including the lack of a thorough description and clearly defined study population sample size, as well as incomplete or ambiguous reporting of recruitment details, and inconsistency in applying the inclusion and exclusion criteria.

This careful documentation allowed us to identify the “strengths and limitations” of the studies (page 28). Notably, the four studies included in this systematic review focused on colorectal cancer patients, with 54% from China and the remaining 46% from various countries, as indicated in Table 1. However, the predominance of data from one geographical area raises concerns about potential geographic bias and the influence of ethnocultural factors, given the likelihood of a singular ethnic, dietary, and cultural background.

To address the reviewer’s question, these differences in recruitment approaches were carefully considered in relation to their impact on the generalizability of the study findings, as also detailed in the “Strengths and Limitations” section (Page 28). We recommended, “To enhance the validity and reliability of findings, future research in this domain must consider a more geographically diverse patient population, addressing these potential biases.” (Page 28-29) This recommendation takes into account the inherent challenges and complexities associated with designing and executing clinical studies involving cancer patients and collecting stool samples while accounting for a wide range of variables.

**Comment 4: The article could provide a comprehensive overview of the role of phyla-based gut microbiota effects in colorectal cancer and its response to immunotherapy.**

**Answer:**

Thank you for your positive feedback on the scope of our manuscript. We appreciate your recognition of our efforts to provide a comprehensive overview of phyla-based gut microbiota effects in colorectal cancer and its response to immunotherapy.

**From Results Section:**

**Comment 1: Can you elaborate on the characteristics of the studies included in the analysis, such as their sample sizes, publication years, and geographic locations?**

**Answer:**

Thank you for your inquiry. We have detailed the sample sizes, publication years, and geographic locations of the studies within the manuscript. Specifically, this information is presented in **Table 1** and described in the text on page 14. To elaborate:

- The studies were published between 2017 and 2022.
- The sample sizes ranged from 60 to 89 participants.
- Cheng et al. 2022, Peng et al. 2020, and Pi et al. 2020 were conducted in China, while the study by Koptez et al. 2017 is part of the international CheckMate 142 clinical trial, which took place across eight countries: Australia, Belgium, Canada, France, Ireland, Italy, Spain, and the United States.

We appreciate your request for clarification, and we believe that the information provided in our manuscript will satisfy the need for a clear and comprehensive understanding of the study characteristics (Page 14).

**Comment 2: How did the studies by Cheng *et al.,* Peng *et al.,* and Kopetz *et al.* differ in their methodologies and findings regarding the relationship between gut microbiome composition and response to immunotherapy in colorectal cancer patients (except the clinical trials)?**

**Answer:**

We appreciate the request for further clarification. These details are outlined in the results section “**Predictive taxonomic profiles of CRC patients in response to ICIs**” on **pages 15-21**. To summarize:

Kopetz et al. observed shifts in the gut microbiome of CRC patients with dMMR and high MSI-H treated with ICIs, identifying lower abundances of *Rothia* and *Rothia mucilaginosa* in partial responders compared to those with progressive disease. This study served as part of the broader CheckMate 142 clinical trial series.

Pi et al. investigated the presence of different bacterial co-abundant gene groups between responders and non-responders to CRC ICIs. Responders with six months of progression-free survival demonstrated a high abundance of *Akkermansia muciniphila*. Conversely, non-responders were found to harbor different bacterial CAGs, including several *Firmicutes* and *Bacteroides* families.

Peng et al. implemented 16S rRNA gene sequencing in advanced-stage gastrointestinal cancer patients to identify differential abundant operational taxonomic units between responders and non-responders to ICIs. They found an abundance of Prevotellaceae, potentially predictive of disease stabilization, and active machine learning techniques identified Firmicutes taxa as top predictors for therapy response. Their research also confirmed a consistent correlation between *Prevotella* and *Bacteroides*.

Cheng et al. conducted a comprehensive study across various types of advanced cancers. They noted distinct differences in gut microbial biodiversity between responders and non-responders before and after ICI therapy, recording an abundance of certain microbes like *Archaea* and *Lentisphaerae* in responders. In their network analysis, they determined the interrelationships among bacterial phyla within the gut microbiota of advanced cancer patients.

These studies, while utilizing similar sequencing techniques, differed in patient populations, sequencing analysis, prediction methods, and the types of ICIs employed; thus, providing a multifaceted picture of how gut microbiota composition might influence immunotherapy’s effectiveness in CRC.

We hope this synthesis of methodologies and findings provides a more comprehensive understanding of the intricate relationship between the gut microbiome and immunotherapy outcomes.

**Comment 3: What were the primary outcomes regarding the predictive taxonomic profiles of colorectal cancer patients in response to immune checkpoint inhibitors (ICIs) based on the included studies?**

**Answer:**

We appreciate your attention to the details of our manuscript. This question is closely related to the previous question that focused on how the studies by Cheng et al., Peng et al., and Kopetz et al. differ in their methodologies and findings.

To reiterate and address the current question, the primary outcomes highlighted in these studies all demonstrated that there are specific taxonomic profiles of microbiota that may predict the effectiveness of ICIs in colorectal cancer patients. The key findings across the included studies are as following:

- Kopetz et al. found that certain taxa, such as *Rothia* and *Rothia mucilaginosa*, were less abundant in CRC patients who showed partial responses to ICIs. These differences in abundance may serve as indicators of patient responses to ICIs.
- Pi et al. and Peng et al. both noted a higher abundance of *Akkermansia muciniphila* in responders, implying a potential predictive role for this bacterium. Peng et al. also found Prevotellaceae to be more abundant in responders than in non-responders.
- Cheng et al. observed that specific microbes such as *Archaea*, *Lentisphaerae*, and other taxa were more abundant in pan-cancer responders prior to therapy, indicating a possible pan-tumor predictive signature for ICI response.

Each of these studies contributes to a growing body of evidence that suggests a significant association between the gut microbiome composition and the clinical efficacy of ICIs in colorectal cancer therapy. Their outcomes suggest that certain microbial signatures could potentially serve as biomarkers for predicting immunotherapy responses.

We have covered these outcomes extensively in the manuscript, particularly in the Results and Discussion sections, which we believe will provide comprehensive insights for the readers.

**Regarding the Bias Section:**

**Comment 1: Can the authors explain the potential sources of bias identified in the included studies, such as methodological and reporting limitations?**

**Answer:**

Thank you for your question. We have evaluated the risk of bias in each study and have provided detailed assessments within our manuscript “**Assessment of risk of bias**” on **pages 23-24**. Here are some key points from our evaluation:

Kopetz et al. and Pi et al. were rated as ‘Fair’ according to the NIH Quality Assessment Tools, which suggests that these studies have certain methodological limitations that could introduce bias. These limitations include a lack of comprehensive descriptions of the study population, ambiguous reporting of recruitment details, and inconsistencies in the application of inclusion and exclusion criteria. Furthermore, the absence of accessible raw data repositories presents an additional challenge, restricting the ability to perform a full and transparent meta-analysis.

On the other hand, Peng et al. received a ‘Good’ rating, indicating that they addressed most of the potential confounding factors at play, which lessens the risk of bias. Cheng et al. conducted an exploratory study and did not apply the usual statistical adjustments to limit the possibility of finding chance associations in the course of making multiple comparisons. The goal of their study was to uncover new trends and generate hypotheses for further research, rather than confirming hypotheses or drawing statistically significant definitive conclusions.

These potential sources of bias have been considered and discussed in our systematic review. We have transparently reported these limitations and considered them when interpreting the results of our systematic review. This assessment ensures that readers can critically evaluate the validity and reliability of the study findings presented.

For further details and the comprehensive risk of bias assessment for each included study, please refer to **Figure 4** and **Table 2** in the manuscript.

**Comment 2: How did the authors address or mitigate the impact of these biases on the internal validity of the systematic review?**

**Answer:**

Thank you for the follow-up question. As outlined in our previous response, we conducted a rigorous evaluation of the risk of bias in each of the included studies using standardized assessment tools. To mitigate the impact of these biases:

- We provided a clear and comprehensive synthesis of the findings, considering the limitations and methodological quality of each study.
- We discussed the implications of these biases in our results and conclusions, ensuring our interpretation of the data is our analysis of the data is impartial and firmly rooted in the framework of these limitations.
- Where possible, we sought to validate key findings through comparisons with the broader literature.
- We refrained from over-interpreting, and generalizing the results from studies with higher potential biases, particularly when the biases could significantly affect the reported outcomes or the conclusions that could be drawn from them. we have proposed additional well-structured clinical research studies to be conducted in the future.

For further mitigation strategies and a detailed discussion on how we accounted for the risk of bias, I would refer you back to our previous answer where we outlined our assessment approach. To reiterate, this evaluation and the steps taken to address the biases are documented in our manuscript (**Pages 23-24**; and **Figure 4** and **Table 2)**.

**Regarding strengths and limitations**

**Comment 1: What were the main strengths of the reviewed studies, and how do they contribute to the understanding of the connection between gut microbiota and immune checkpoint inhibitors (ICIs)?**

**Answer:**

Thank you for your question. The reviewed studies are strong in their rigorous methodologies (highlighted in the usage of advanced molecular techniques to investigate the gut microbiome) and significant in their exploration of the connection between gut microbiota and ICIs. By identifying specific bacterial species associated with patient responses to ICIs, these studies contribute to the field of cancer treatment, offering foundational insights for future research. The consistent identification of certain bacterial species, such as *Akkermansia* and *Bifidobacterium*, as predominant in patients exhibiting favorable responses to immunotherapy underscores the viability of utilizing gut microbiota composition as prognostic biomarkers This lays the groundwork for more personalized approaches to cancer treatment.

Please refer to “Strengths and limitations” in the manuscript (**Pages 28-29**).

**Comment 2: How might the limitations of the included studies, such as small sample sizes and geographic bias, impact the generalizability of the findings and the ability to draw comprehensive conclusions?**

**Answer:**

We appreciate the question. The limitations of the included studies, particularly small sample sizes and geographic bias, raise concerns about the generalizability of the findings. The concentration of data from primarily one geographical location prompts questions about the broader applicability of the results across different populations with diverse ethnic and cultural backgrounds. To improve the generalizability of these findings, we recommended in the systematic review that subsequent research must incorporate larger and more varied sample sizes from multiple geographic regions. This strategy will facilitate the validation and potential expansion of the relevance of the existing findings to diverse populations.

Please refer to “Strengths and limitations” in the manuscript (**Pages 28-29**).

**Comment 3: How did the studies address potential limitations in data interpretation, such as the dynamic nature of the microbiome and the variability in microbial composition among individuals?**

**Answer:**

Thank you for the question.

The dynamic nature of the gut microbiome and individual variability present challenges that were not fully addressed in most of the included studies, as they primarily relied on baseline profiling. However, Cheng et al. conducted longitudinal (baseline and follow-up) assessments to track changes in the microbiome throughout ICI treatments. This continuous evaluation provides a deeper understanding of the microbiota’s role during treatment, potentially linking specific changes to treatment outcomes. Despite the limitation, methodologies that take into account the dynamic nature of the microbiome may provide enhanced understanding of the correlation between microbial makeup and response to immune checkpoint inhibitors (ICIs). These approaches are advocated for in forthcoming research endeavors, as highlighted in the systematic review. Please refer to “Strengths and limitations” in the manuscript (**Pages 28-29**).

**Regarding future clinical relevance**

**Comment 1: Based on the findings of the included studies, what implications do these results have for the development of personalized treatment strategies for colorectal cancer patients receiving immunotherapy?**

**Answer:**

Thank you for the insightful question.

The findings from the included studies suggest a potential path forward for personalized treatment strategies in colorectal cancer immunotherapy. By mapping an individual’s gut microbiota composition and assessing bacterial activity (particularly the production of beneficial short-chain fatty acids like acetate, propionate, and butyrate) which have been associated with positive responses to immune checkpoint inhibitors, clinicians might be able to predict and optimize the efficacy of ICI therapy. For instance, the presence of microbial taxa such as *Lachnospiraceae* and bacteria possessing the ability to produce short-chain fatty acids (SCFAs) have the potential to not only contribute to meeting the energy needs of immune cells but also play a role in boosting the body's response against tumor cells. As a result, profiling the microbiome for these functional capabilities could become an integral part of developing a tailored approach to immunotherapy treatment for colorectal cancer patients, potentially improving outcomes by aligning treatment with individual microbial biomarkers.

Please refer to the “Discussion” (**Pages 26-27**) and “Conclusion” (**Page 30**) sections.

**Comment 2: Were there any consistent patterns or trends observed across the studies that could inform clinical decision-making regarding the use of immunotherapy in colorectal cancer patients with specific microbiome profiles?**

**Answer:**

Thank you for prompting further exploration on this topic.

The findings from multiple studies identifying consistent associations between certain gut microbiota, such as the abundance of microbial families like *Lachnospiraceae*, *Akkermansia* *municiphila*, and the production of SCFAs, and positive responses to ICIs, suggest the promising potential of gut microbiome profiling in informing personalized treatment strategies for colorectal cancer. The observed link between favorable immunotherapy outcomes and specific bacterial activity (including the production of beneficial SCFAs like acetate, propionate, and butyrate) underscores a functional aspect of the microbiome that may contribute to the clinical efficacy of ICIs. This relationship presents an opportunity to develop predictive models that determine the potential responsiveness to immunotherapy based on the composition and metabolic functions of an individual’s gut microbiota. Such an approach could tailor ICI therapy, thus optimizing the treatment for CRC patients and possibly enhancing the therapeutic outcomes.

Please refer to the “Discussion” (**Pages 26-27**) and “Conclusion” (**Page 30**) sections.

**Comment 3: How might the identification of predictive taxonomic profiles for immunotherapy response in colorectal cancer patients impact future research and clinical practice in oncology?**

**Answer:**

Thank you for your inquiry.

The answer to this inquiry will mirror the insights provided earlier, as both questions delve into the related theme of microbial patterns and their implications for clinical practice.

The patterns seen across the studies consistently show that certain gut microbiota such as Lachnospiraceae, *Akkermansia municiphila*, as well as those involved in SCFA production like *Akkermansia* and *Bifidobacterium*, have a potential association with better responses to immune checkpoint inhibitors. These findings can guide clinicians in identifying which colorectal cancer patients might benefit most from immunotherapy, suggesting that microbiome profiling could become a valuable tool in optimizing immunotherapy regimens based on specific microbiome compositions.

Please refer to the “Discussion” (**Pages 26-27**) and “Conclusion” (**Page 30**) sections.

**Comment 4: Are there specific aspects of the gut microbiome or immunotherapy response that were not adequately addressed by the included studies, and if so, what areas warrant further investigation?**

**Answer:**

Thank you for your insightful question.

The reviewed studies highlight promising correlations between gut microbiota and responses to ICIs, yet there remain specific aspects that necessitate deeper exploration. The potential regulatory function of relatively unexplored bacteria such as *Parasutterella* in ICI response has not received extensive scrutiny. Moreover, the interplay among different intestinal microbial profiles, the metabolites they generate, and various immunotherapeutic agents demands additional exploration. Apart from delineating microbial taxa linked to successful outcomes in immunotherapy, there is a necessity to probe into microbial activities associated with lack of response to immunotherapy. For example, the impacts of heightened lipopolysaccharide biosynthesis and sugar degradation on the intestinal milieu and their subsequent effects on antitumor immune responses are areas that necessitate more extensive inquiry. Additionally, could enhancing the presence of these specific bacteria enhance the efficacy of ICI response? Understanding these nuances in microbial metabolism and function could shed light on underlying mechanisms and lead to more effective modulation of the gut microbiome to enhance immunotherapy efficacy.

Please refer to the “Discussion” (**Pages 26-27**) and “Conclusion” (**Page 30**) sections.

**Comment 5: How might advances in technology and methodology, such as multi-omics approaches or longitudinal studies, enhance our understanding of the complex interplay between the microbiome and cancer immunotherapy outcomes?**

**Answer:**

Thank you for your comment.

Advances in technology and methodology, particularly by multi-omics approaches, are poised to significantly enhance our understanding of the intricate relationship between the microbiome and cancer immunotherapy outcomes. By improving the characterization of complex microbiota and their functional potential with high-resolution and comprehensive analytical tools, such as SILVA, GreenGenes, and MetaPhlAn2, along with the metabolic profiling tools, researchers can delve deeper into the microbiota’s contributions to ICI efficacy for instance, it could aid in the identification of specific metabolites as potential biomarkers or pharmaceutical agents. The precision and standardization afforded by these advancements will allow for a more accurate assessment of microbial communities and their dynamic changes during treatment.

Longitudinal studies, which track these variations over time, will be especially valuable in shedding light on time-sensitive microbial signatures that correlate with treatment outcomes. Together, these methodological improvements promise to reveal a more nuanced understanding of the microbiome’s role in modulating the immune response during cancer immunotherapy, potentially leading to more personalized treatment strategies and better patient outcomes.

Please refer to the “Discussion” (**Pages 26-27**) and “Conclusion” (**Page 30**) sections.

Thank you for your consideration of this revised manuscript. We hope that these changes will satisfy the reviewers’ expectations and that the manuscript finally finds a place in your reputable journal.

Yours Sincerely,

Zakeya Al Rasbi
